# Activation of adult mammalian retinal stem cells in vivo via antagonism of BMP and sFRP2

**DOI:** 10.1186/s13287-021-02630-0

**Published:** 2021-10-30

**Authors:** Kenneth N. Grisé, Brenda L. K. Coles, Nelson X. Bautista, Derek van der Kooy

**Affiliations:** 1 Department of Molecular Genetics, University of Toronto, Donnelly Centre Rm 1110, 160 College Street, Toronto, ON M5S 3E1 Canada; 2 Institute of Medical Science, University of Toronto, Toronto, ON M5S 1A8 Canada

**Keywords:** Retinal stem cells, Stem cells, Ciliary epithelium, Retina, Retinogenesis, Proliferation, Regeneration, Adult stem cells, Niche, Ciliary marginal zone, Vision, Eye

## Abstract

**Background:**

The adult mammalian retina does not have the capacity to regenerate cells lost due to damage or disease. Therefore, retinal injuries and blinding diseases result in irreversible vision loss. However, retinal stem cells (RSCs), which participate in retinogenesis during development, persist in a quiescent state in the ciliary epithelium (CE) of the adult mammalian eye. Moreover, RSCs retain the ability to generate all retinal cell types when cultured in vitro, including photoreceptors. Therefore, it may be possible to activate endogenous RSCs to induce retinal neurogenesis in vivo and restore vision in the adult mammalian eye.

**Methods:**

To investigate if endogenous RSCs can be activated, we performed combinatorial intravitreal injections of antagonists to BMP and sFRP2 proteins (two proposed mediators of RSC quiescence in vivo), with or without growth factors FGF and Insulin. We also investigated the effects of chemically-induced *N*-methyl-*N*-Nitrosourea (MNU) retinal degeneration on RSC activation, both alone and in combination withthe injected factors. Further, we employed inducible *Msx1-Cre*^*ERT2*^ genetic lineage labeling of the CE followed by stimulation paradigms to determine if activated endogenous RSCs could migrate into the retina and differentiate into retinal neurons.

**Results:**

We found that in vivo antagonism of BMP and sFRP2 proteins induced CE cells in the RSC niche to proliferate and expanded the RSC population. BMP and sFRP2 antagonism also enhanced CE cell proliferation in response to exogenous growth factor stimulation and MNU-induced retinal degeneration. Furthermore, *Msx1-Cre*^*ERT2*^ genetic lineage tracing revealed that CE cells migrated into the retina following stimulation and/or injury, where they expressed markers of mature photoreceptors and retinal ganglion cells.

**Conclusions:**

Together, these results indicate that endogenous adult mammalian RSCs may have latent regenerative potential that can be activated by modulating the RSC niche and hold promise as a means for endogenous retinal cell therapy to repair the retina and improve vision.

**Supplementary Information:**

The online version contains supplementary material available at 10.1186/s13287-021-02630-0.

## Background

The pigmented ciliary epithelium (CE) in the adult mammalian eye harbours a rare subpopulation of retinal stem cells (RSCs) that are capable of clonal expansion, self-renewal, and differentiation into all the cell types of the retina when isolated in vitro. [1–6]. The CE is an epithelial bilayer that overlays the ciliary body and is contiguous with the peripheral retina and retinal pigmented epithelium (RPE) [7]. The anatomical location and properties of CE-RSCs has led to their comparison with the stem cells in the ciliary marginal zone (CMZ) of non-mammalian vertebrates, which have the capacity for retinal neurogenesis and regeneration in response to injury throughout the lifespan of the organism [8, 9]. However, unlike stem cells in the CMZ, CE-RSCs become quiescent in the early postnatal period and remain dormant in vivo throughout maturity [10–13]. Indeed, it is typically held that the adult mammalian RSC niche does not respond to exogenous stimulation or injury, and does not have neurogenic potential in vivo [9–14].

Although CE-RSCs and their progeny have been shown to express stem cell and retinal progenitor genes, some studies have reported that RSCs have limited in vitro proliferative/self-renewal ability, that RSC progeny maintain features of epithelial cells, and described only ectopic expression of mature retinal cell markers after differentiation [15, 16]. This led to the suggestion that CE cells might have general proliferative competency and plasticity as opposed to containing rare stem cells. However, more recent experiments have shown that RSCs can be prospectively identified and sorted [5], that in vitro growth and self-renewal of RSCs and their progeny can be profoundly enhanced based on cell culture conditions [2, 17], and that RSC-derived progenitors can generate functional photoreceptors in vitro [18, 19] and in vivo following transplantation [3, 20]. This supports the existence of a rare cell type within the CE with proliferative competency and corroborates the neurogenic capacity of adult RSCs. Furthermore, recent in vivo lineage tracing studies have revealed that the mammalian CE contributes to retinogenesis during development, when CE cells migrate into the retina and generate all seven major retinal cell types [21, 22]. Together, these findings have led to the hypothesis that, following development, inhibitory factors arise in the mammalian CE niche that maintain RSCs in a quiescent state and prevent their stimulation, proliferation and differentiation into new retinal neurons [12, 14, 23].

A previous study by our lab discovered that Bone Morphogenetic Proteins (BMP) 2 & 4 and secreted frizzled-related protein 2 (sFRP2) are secreted by the adult lens and cornea and are capable of suppressing RSC proliferation and clonal RSC-derived sphere growth in vitro [23]. BMP and Wnt signaling pathway components were shown to be present in both RSC spheres and the CE. Further, BMP and sFRP2 proteins were shown to modulate BMP and Wnt signaling activity, respectively, in RSC spheres. When BMP antagonist, Noggin, or an anti-sFRP2 function blocking antibody was added to lens and cornea conditioned media, RSC proliferation inhibition was reversed. BMP2 and BMP4 proteins are expressed in the developing CE and are required for normal CE development and morphogenesis [24, 25]. sFRP proteins are well-known as Wnt antagonists [26–28] and Wnt signaling is required for specifying the CE during development [29–31] and can modulate the number of RSCs in vivo [32]. In addition, BMP and Wnt signaling are known to regulate retinal stem and progenitor cell proliferation and differentiation in the non-mammalian CMZ [33, 34]. Thus, we hypothesized that BMP2/4 and sFRP2 may be the main inhibitory factors in the adult eye that mediate RSC quiescence [23].

In this study we show that intravitreal injection of Noggin or anti-sFRP2 induces proliferation of cells in the CE and expands the RSC population in vivo. Using the inducible *Msx1-Cre*^*ERT2*^ mouse line, we lineage label the adult CE and reveal that RSCs and RSC-derived progenitors are also labeled. Further, by lineage labeling the CE, we show that combinatorial injection of noggin, anti-sfrp2, FGF2 and insulin, with or without retinal injury, induces CE cells to migrate into the retina where they express markers of mature photoreceptors or retinal ganglion cells.

## Methods

### Mice

All mouse protocols were approved by the Animal Care Committee at the University of Toronto, which operates in accordance with the Canadian Council on Animal Care. Mice were kept on a 12-h light dark/light cycle. Food was available ad libitum. Water was supplied ad libitum except during EdU delivery (see below). The number of mice used in each experiment is indicated in the figure captions. Adult mice used in this study were 8–24 weeks old and included: CD1 mice (022, Charles River), C57/BL6J mice (000,664, Jackson Laboratories), B6.Cg*-Gt(Rosa26)Sor*^*Tm14(CAG−tdTomato)Hze*^ mice [35] (007,914, Jackson Laboratories) and *Msx1-Cre*^*ERT2*^ mice [36]. The *Msx1-Cre*^*ERT2*^ mouse line was a gift from Dr. Michel Cayouette and Dr. Benoît Robert and the CreERT2 construct originated from the IGBMC via Pierre Chambon. *Msx1-Cre*^*ERT2*^ is a transgenic line where the CreERT2 fusion protein is expressed in place of the endogenous Msx1 protein, as it has been knocked in, in frame, in the first exon of *Msx1*. *Msx1-Cre*^*ERT2*^ mice were crossed with B6.Cg*-Gt(Rosa26)Sor*^*Tm14(CAG−tdTomato)Hze*^ mice to generate *Msx1-Cre*^*ERT2*^;B6.Cg*-Gt* (Rosa26*)Sor*^*Tm14(CAG−tdTomato)Hze*^ mice, which were used for lineage tracing experiments. Each mouse used was genotyped by PCR amplification. For full list of genotyping primers see Additional file 1.

### Drug and protein preparations

EdU (Thermo; A10044) was dissolved at 0.2 mg/mL in 1% sucrose (BioShop; SUC507.1) in ddH_2_O and placed into 50 mL Falcon tubes fitted with standard rubber water stoppers/sipper tubes for ad libitum access during intravitreal injection periods. The EdU water level was topped up daily. Systemic administration of EdU in the drinking water has been shown to elicit consistent EdU labeling in dividing cells at this dose [37, 38].

Tamoxifen (Sigma; T5648) was dissolved in 1:10 anhydrous EtOH (Sigma; 676,829) to sunflower seed oil (Sigma; S5007), vortexed and incubated for 15 min in a 37 °C water bath. It was injected i.p. at 180 mg/kg, once a day for 4 days, in order to induce reporter expression in *Msx1-Cre*^*ERT2*^;B6.Cg*-Gt* (Rosa26*)Sor*^*Tm14(CAG−tdTomato)Hze*^ mice.

CHIR99021 (Sigma; SML1046) was dissolved dissolved in DMSO (Sigma; 276,855) and then diluted to 0.525 µM or 5.25 µM to arrive at 1.75% DMSO in PBS, which was then injected into the 7µL intravitreal space [39] to arrive at a final in vivo concentration estimated at 0.15 µM or 1.5 µM, respectively, in 0.5% DMSO. LDN-193189 (SelleckChem; S2618) was dissolved dissolved in DMSO and then diluted to 0.35 µM or 3.5 µM to arrive at 1.75% DMSO in PBS, which was then injected into the 7µL intravitreal space to arrive at a final in vivo concentration estimated at 0.1 µM or 1 µM, respectively, in 0.5% DMSO.

Noggin (R&D Systems; 719-NG-050), anti-sFRP2 (R&D Systems; MAB1169), FGF2 (100 ng/eye; R&D Systems; 3139-FB-025/CF) and Insulin (2 µg/eye; Sigma; 16,634) were dissolved directly in PBS. All concentrations listed for proteins in the manuscript are the final in vivo concentrations accounting for the 7µL intravitreal space.

### Intravitreal injections

Intravitreal injections were carried out using a 10µL WPI Nanofil® Injector System with micro-machined 34-gauge beveled needle (World Precision Instruments, Sarasota, FL), a dissecting microscope or surgical scope (Moller Hi-R 900C), a mouse stereotaxic apparatus and heat pad. Mice were brought to a surgical plane of anesthesia via 5% isoflurane and placed on the heat pad in the mouse stereotaxic apparatus (without head stabilization with the ear bars). Once anesthetized, isoflurane was reduced to 3% for maintenance. Mice were injected with 2 mg/Kg meloxicam for analgesia. One drop of anticholinergic mydratic (Mydriacyl®) was applied to each mouse eye to dilate pupils. Mice were positioned on one side, so that the eye to be operated on was facing upward, directly under the surgical microscope. A small rubber washer was placed over the eye, so that the washer surrounds the eye like a monocle. A single drop of 3% methylcellulose (MC) solution (in saline) into the monocle, which allows clear visualization of the posterior segment of the eye by the surgeon. The mouse head was stabilized with the non-dominant hand and the needle was controlled with the dominant hand. With the needle, a trans-scleral puncture was made (at a perpendicular angle to the globe) approximately 1 mm posterior of the limbus, in the nasal (anterior) aspect of the eye. The needle passed through the sclera, choroid and retina to enter the retrolental vitreous. The needle was inserted as far as the central area of the retina, taking care to avoid striking the lens, retina or the hyaloid canal. A 2µL bolus of fluid was then injected at an approximate rate of 4µL/min. The adult mouse vitreous space can accommodate up to 3 ul of total fluid because it replaces fluid initially lost from pre-injection vitreous outflow. Thus, the final vitreous volume in the eye is the same as the standard vitreous volume of the mouse eye (7µL) [39]. Once the injection was completed, the needle remained in the retrolental vitreous for an additional 10–15 s. This allows for pressure equilibration and works to prevent significant backflow following withdrawal of the needle. Next, the needle was removed, the monocle was removed, and the mouse is rotated to position the other eye for surgery. Once the surgery on both eyes was completed, the mouse was left to recover alone in a recovery cage with a heat lamp, and then reunited with its original cage-mates.

### Immunohistochemistry

Mice were euthanized by cervical dislocation while under isoflurane anesthesia. Eyeballs were enucleated from adult mouse skulls, post-fixed in 4% PFA for 4 h at 4 °C, then transferred to a cryoprotectant 30% sucrose solution for a minimum of 24 h at 4 °C. Next, eyes were embedded in Tissue Tek, frozen at -80 °C and then sectioned at 10 µm using a cryostat. Fixed frozen eye slides were permeabilized with 0.3% Triton X-100 (Sigma; T8787) in PBS for 20 min. Then, they were blocked in 10% normal goat serum (NGS) or 10% normal donkey serum (NDS) for 1 h. Primary antibodies were diluted in 1% serum from the species used for blocking (to the dilutions indicated below) and incubated overnight at 4 °C. After washing, secondary antibodies were diluted in 1% serum of the same species at 1:400 (Alexa fluor; ThermoFisher) and incubated for 1 h. After washing, nuclei were stained with Hoechst 33,258 (10 μg/mL; ThermoFisher; H1399) or DRAQ5 (1uM; ThermoFisher; 62,251) for 20 min before a final wash. Mounting medium was added to wells or slides and slides were then coverslipped. Primary antibodies used in this study are listed in the Key Resources Table and were used at the following dilutions: rabbit anti-Ki67 (1:100; Ab15580; Abcam), rabbit anti-Recoverin (1:1000; AB5585; Millipore), goat anti-Chx10 (1:1000; Sc-21690; Santa Cruz), rabbit anti-cone arrestin (1:1000; AB15282; Millipore), mouse anti-rhodopsin (1:500; MAB5316; Millipore), goat anti-Brn3a (1:500; SC-31984; Santa Cruz) rabbit anti-Pax6 (1:1000; AB2237; Millipore), rabbit anti-ERG (1:250; AB92513; Abcam) and rat anti-CD68 (1:500; MCA1957; BioRad).

### Imaging and cell type quantification

Slides were imaged using either a Zeiss Axiovert inverted microscope (with Zeiss Axiovision v4.8.2) or Olympus FV1000 confocal microscope (with Olympus Fluoview FV10-ASW v4.2b). Images were analyzed using Image J software (http://rsbweb.nih.gov/ij/). The CE is identifiable as a cuboidal cell, epithelial bi-layer on the inner surface of the ciliary body which extends between the iris and retina (Additional file 1: Figure S1). To analyze the CE, images were loaded into Image J, then “set scale” to the image-embedded scale bar, and the “freehand” drawing tool was used to trace the CE region and calculate the CE area (see Additional file 1: Figure S1D for example of freehand tracing). Cells types of interest were counted and divided by CE area to normalize across sections. The anterior CE-iris border is readily apparent by morphology. The finger-like ciliary processes of the pars plicata transition at the iris root to become the linear, pigmented iris epithelial bilayer of rectangular cells, which extend anteriorly at a variable angle from the CE (Additional file 1: Figure S1A-B). The anterior pigmented epithelial layer of the iris is continuous with the outer pigmented epithelial (OPE) layer of the CE, while the posterior pigmented epithelial layer of the iris is continuous with the inner non-pigmented epithelial (NPE) layer of the CE. At the posterior border of the CE, the OPE layer of the CE is continuous with the RPE, and the inner NPE layer is continuous with the NR. Where the CE-NR transition occurs, the single cell layer of the NPE expands into the multi-layered laminated retina, with dense nuclei in the outer nuclear layer (ONL) where the photoreceptors reside. Photoreceptor-specific markers, such as recoverin, are an excellent aide to delineate this transition (Additional file 1: Figure S1C).

### Isolation of retinal stem cells from the ciliary epithelium of the adult eye and primary clonal sphere assay

A dissecting microscope, cold light source, and sterile surgical instruments were set up inside of a sterile biological safety cabinet (BSC). Mouse eyes were enucleated immediately prior to beginning the dissection protocol. Mouse or human eyes were placed in a petri dish containing cold, sterile, artificial cerebrospinal fluid (aCSF). Under the dissecting microscope, hair, connective tissue and the dorsal and ventral oblique muscles were cleared from the scleral/corneal border with two sets of forceps. Next, curved or angled micro-dissecting scissors were used to cleave off any remaining extraocular muscle tissue and the optic nerve and cut the eyeball into symmetrical halves, beginning and finishing the cut from the hole left by the optic nerve. Using two sets of forceps to grasp the cornea, the two eye halves were peeled apart. The lens, retina, and vitreous were separated from the eye shells and the eye shells were transferred into a new petri dish (also containing cold, sterile aCSF). To isolate the ciliary epithelium (CE), eye shells were oriented with the cornea on the right and retinal pigmented epithelium (RPE) on the left. A pair of straight forceps were used to pin down the eye shell on the RPE side while a scalpel blade was inserted between the CE and the iris, using pressure to slice the iris/cornea side off from the rest of the shell. Next, the scalpel was run along the border between the CE and the RPE to obtain the CE isolated as a thin strip of tissue. The CE strips were then transferred to a 35 mm dish containing 2 mL of dispase solution (Sigma; T1005) and incubated for 10 min at 37 °C. Next, the strips were transferred from dispase into a 35 mm dish containing 2 mL of sterile filtered kynurenic acid (02.mg/mL; Sigma), trypsin (1.33 mg/mL; Sigma) and hyaluronidase (0.67 mg/mL; Sigma) in high magnesium/low calcium aCSF (hi/lo aCSF) and incubated at 37 °C for 10 min. After incubation, the dish was returned to the dissecting scope, and the CE strips were pinned down with straight, non-serrated forceps, while non-serated curved forceps were used to scrape the CE off from the underlying sclera. The bare scleral strips were then discarded, such that only the CE cells remained in the enzyme solution. Using a fire-polished, cotton-plugged glass pipette, the cells and enzyme solution were transferred to a 15 mL tube and triturated approximately 45 times to break apart the tissue. The 15 mL tube with cell suspension was centrifuged for 5 min at 1500 rpm. The supernatant was gently aspirated from the resulting pellet using a fire-polished, cotton-plugged glass pipette and 2 mL of sterile-filtered ovomucoid trypsin inhibitor (1 mg/mL; Sigma) in serum-free media (SFM) was added to the pellet. Using a small borehole, fire-polished, cotton-plugged glass pipette, the sample was triturated approximately 45 times to generate a single-cell suspension. The 15 mL tube with cell suspension was centrifuged for 5 min at 1500 rpm. The supernatant was gently aspirated from the resulting pellet and 1-2 mL of SFM with fibroblast growth factor 2 (FGF2, 10 ng/mL; Sigma) and heparin (2 μg/mL; Sigma) (SFM + FH) was added. The cells and media were mixed to ensure a uniform cell suspension and a 10µL sample was taken for cell density determination. The cells were then seeded and cultured at 10 cells/μL in culture-treated plates or flasks and incubated in a humidified incubator at 37 °C in 5% CO_2_ and ambient room O_2_. After one week, roughly 1 in 500 cells are expected to proliferate to form free-floating, clonal spheres greater than 80 μm in diameter.

### Fluorescence assisted cell sorting

The ciliary epithelium was dissected dissociated to single cells as outlined in the above section “Isolation of Retinal Stem Cells from the Ciliary Epithelium of the Adult Eye and Primary Clonal Sphere Assay”. The single cell suspension was filtered through a 40 µm cell strainer. Cells were counterstained with DAPI (0.1 µg/mL; Invitrogen; D1306) to assess viability. The CE from from C57/BL6J mice was used to set the gating for pigmented and non-pigmented CE, based on forward and side scatter, and to gate negative tdTomato (red) fluorescence using a FACS Aria II (BD Biosciences). The single cell suspension from the CE of tamoxifen-treated *Msx1-Cre*^*ERT2*^;B6.Cg*-Gt* (Rosa26*)Sor*^*Tm14(CAG−tdTomato)Hze*^ mice was sorted into pigmented and non-pigmented fractions, and also fluorescent and non-fluorescent fractions using endogenous tdTomato expression. After sorting, cells were plated at 1 cell/μL in 500μL of SFM + FH per well in 24-well plates for a 7-day sphere assay. Flow cytometry analysis was performed using BD FACS Diva Software V6.1.2.

### Statistical analysis

Data are presented as mean ± standard error (SE) unless otherwise noted. Statistical analyses were run using Sigmaplot 12 (Systat Software Inc.) or GraphPad Prism 6 (GraphPad Software Inc.). Student’s t-test (two-tailed) was performed for statistical analysis between two groups. A one-way ANOVA or two-way ANOVA with a Holm-Sidak post-hoc test (pairwise or versus control comparison) was used when three or more groups were compared. Sample size (N) and p-values are provided in the figure legends. Statistical significance was set at *p* < 0.05.

## Results

### Noggin or anti-sFRP2 stimulates ciliary body-specific proliferation and expands the retinal stem cell population

**Fig. 1:**
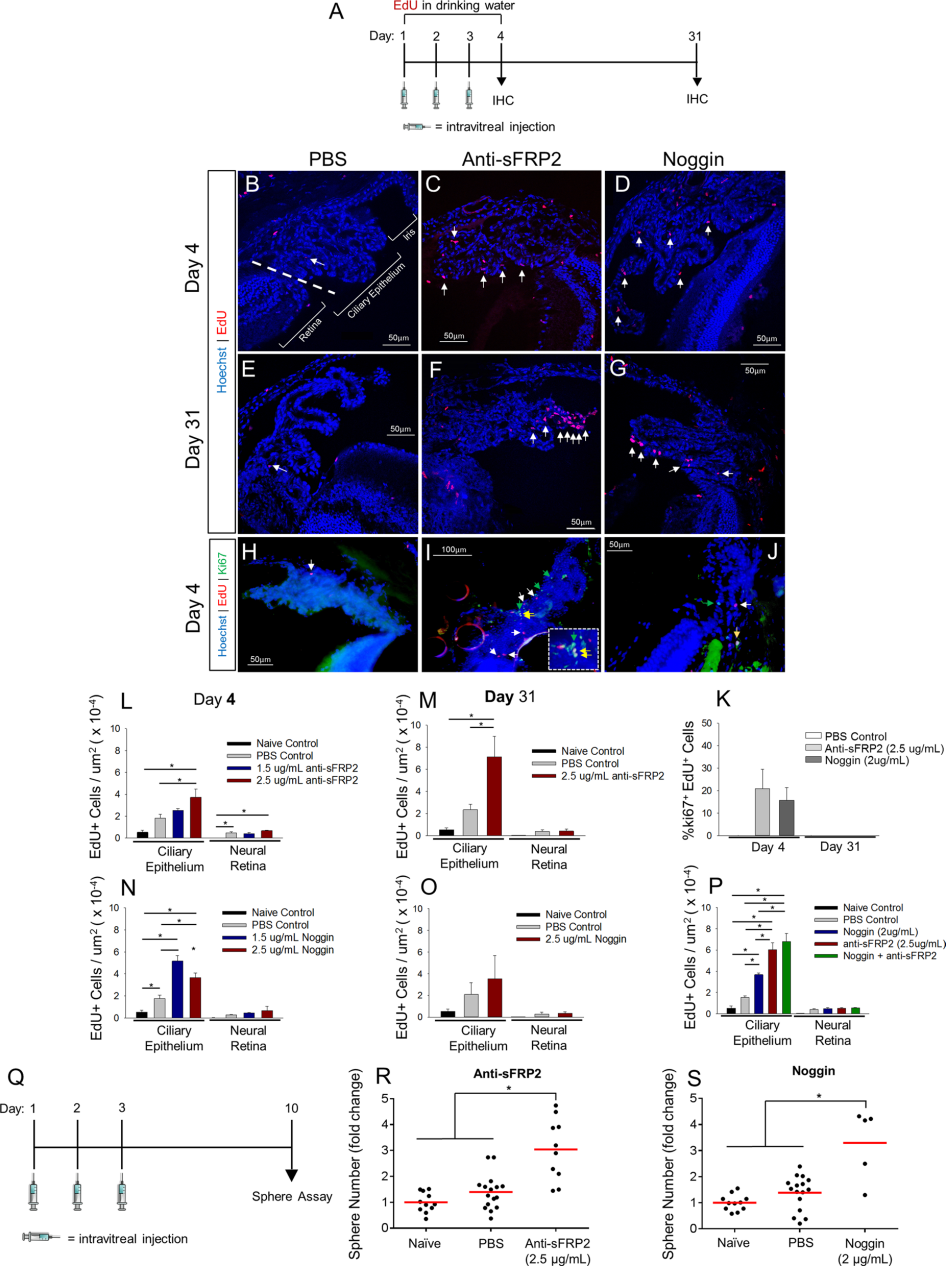
Intravitreal injection of Noggin or anti-sFRP2 stimulates proliferation in the ciliary epithelium and increases primary sphere-forming retinal stem cell number. **A** Schematic of the intravitreal injection paradigm followed by endpoint IHC. Mice received one intravitreal injection per day for three days while EdU was delivered via the drinking water continuously until Day 4. Mice were euthanized for histochemical analysis on Day 4 or Day 31. Injections consisted of PBS control, Noggin or anti-sFRP2 at indicated concentrations. **B-G** Fluorescence images of EdU labeling in the CE of eye sections from mice treated with PBS, anti-sFRP2 or Noggin at Day 4 and Day 31. **B, E** EdU labeling in PBS-treated eyes. **C, F** EdU labeling in Noggin-treated eyes. **D, G** EdU labeling in and anti-sFRP2-treated eyes. Hoechst stain was used to label all nuclei. White arrows indicate EdU^+^ cells. Straight dashed line indicates the border between the ciliary epithelium and the retina. **H-J** Ki67 immunostaining and EdU labeling at the Day 4 timepoint in the CE of eye sections from mice treated with PBS, anti-sFRP2 or Noggin. **h** PBS-treated eyes. **i** Anti-sFRP2-treated eyes. **j** Noggin-treated eyes. White arrows indicate EdU^+^ cells. Green arrows indicate Ki67^+^ cells. Yellow arrows indicate EdU + Ki67 co-labeled cells. Hoechst stain was used to label all nuclei. Dashed line box indicates high magnification inset. **K** Quantification of the proportion of EdU + Ki67 co-labeled cells relative to the total number of EdU-labeled cells in the CE at Day4 and Day 31. N = 3–5 mice per group. **L-O** Quantification of EdU-labeled cells in the CE and NR normalized by regional area in eye sections from mice treated with the indicated conditions. **l** Day 4 anti-sFRP2. (*p* = 0.004; N = 3–6 mice per group). **M** Day 31 anti-sFRP2. (*p* = 0.015; N = 3 mice per group). **N** Day 4 Noggin. (*p* =  < 0.001; N = 3–6 mice). **o** Day 31 Noggin; N = 3 mice per group. **P** Quantification of EdU-labeled cells in the CE and NR normalized by area in eyes treated with PBS, Noggin, anti-sFRP2 or both Noggin + anti-sFRP2 at Day 4. (*p* < 0.001; N = 3–6 eyes per group). **Q** Schematic of the intravitreal injection paradigm followed by endpoint clonal RSC sphere forming assay from primary CE. Mice received one intravitreal injection per day for three days. On Day 10, mice were enucleated, and the CE was dissected for a subsequent 7-day clonal sphere growth assay in vitro. **R, S** Quantification of RSC sphere frequency normalized to naïve un-injected control. **R** anti-sFRP2 dose–response. (*p* < 0.0001; N = 10–16 eyes per group). **(S)** Noggin dose–response. (*p* < 0.001; N = 5–16 eyes per group). Each data point represents a single eye. Statistics: One-way ANOVAs with Holm-Sidak posthoc tests. Data are means ± SEMs. * = * p* < 0.05

To determine if blocking endogenous BMP proteins or sFRP2 proteins present in the adult mouse eye can modulate the proliferation of cells within the CE or the neural retina (NR), we delivered the BMP antagonist, Noggin, or a function blocking anti-sFRP2 antibody in vivo via intravitreal injection (Fig. 1A). Mice received one injection per eye, per day, for 3 consecutive days (2µL volume per injection). For the first four days of the experiment the thymidine analog 5-ethynyl-2’-deoxyuridine (EdU), which is incorporated into the DNA of cells during the S phase of the cell cycle [40], was delivered via the drinking water (0.2 mg/mL). Exploratory dose–response experiments were used to identify concentrations of the injected factors that appeared to increase EdU labeling, which were then used in subsequent experiments. Of note, higher doses were not necessarily more effective, indicating that total protein injected was not responsible for observed proliferation effects. Also, no morphological differences in the CE were observed across any treatment groups described in this experiment. At Day 4 and Day 31 post-inection, anti-sFRP2 treatment (1.5 µg/mL or 2.5 µg/mL) resulted in a discernible increase in EdU^+^ cells in the CE (Fig. 1C,F) relative to PBS control (Fig. 1B,E). At Day 4, EdU^+^ cells were located throughout the CE, usually as single or doublet cells, in both the inner and outer CE layers. At Day 31, larger clusters of EdU^+^ cells were evident. Naïve, un-injected eyes were also examined to assess any effects of PBS vehicle injection on EdU labeling. At Day 4, the 2.5 µg/mL dose of anti-sFRP2 increased EdU labeling in the CE nearly twofold relative to PBS (Fig. 1L). In contrast, neither dose of anti-sFRP2 increased EdU^+^ cell number in the NR, though a small effect of PBS injection versus naïve control was observed. At Day 31, anti-sFRP2 resulted in a ~ fourfold increase in EdU^+^ cells relative to PBS control (Fig. 1M). These results suggest anti-sFRP2 antibody binding and antagonism of sFRP2 proteins in the vitreous results in dose-dependent, CE-specific proliferation.

Intravitreal Noggin treatment (1.5 µg/mL or 2.5 µg/mL) resulted in a discernible increase in EdU^+^ cells in the CE at both Day 4 and Day 31 (Fig. 1D,G). The pattern of EdU^+^ cell localization in the CE after Noggin treatment was similar to anti-sFRP2 treatment, with single and doublet cells evident in both layers of the CE at Day 4, and larger cell clusters observed at Day 31. Quantitatively, at Day 4, each dose of Noggin increased EdU labeling in the CE by roughly threefold and twofold, respectively, relative to PBS control (Fig. 1N). Also, there was an effect of PBS injection versus naïve control in the CE. In contrast, there was no effect of PBS or Noggin on EdU labeling in the NR. Though there was a trend toward increased EdU^+^ cell number in the CE and NR after Noggin injection at Day 31, it was not statistically significant (Fig. 1O). These results indicate that Noggin-mediated antagonism of BMP proteins induces CE-specific proliferation. The non-significant level of EdU labeling in the CE at Day 31 after Noggin treatment could have resulted from attenuated EdU + cell numbers resulting from EdU^+^ cell death, cell migration out of the CE, or possibly, dilution of the EdU label by persistent proliferation [41].

To examine the proportion of EdU^+^ cells that continue to proliferate following the intravitreal injection period, we immunostained for the proliferation marker Ki67. At Day 4, both Ki67^+^ cells and EdU^+^Ki67^+^ co-labeled cells were detected in anti-sFRP2 and Noggin treated eyes, but not in PBS controls (Fig. 1H-J). This demonstrates that a subset of CE cells were still proliferating 24 h after the injection period. However, a low proportion of EdU^+^ cells co-labeled with Ki67 (~ 18–20%), indicating the majority of EdU^+^ cells labeled during the injection period are no longer dividing 24 h later (Fig. 1K). At Day 31, no Ki67 was detectable in any condition; thus, proliferation in the CE does not persist one month after intravitreal injection.

To determine if antagonism of sFRP2 and BMPs has an additive effect on EdU labeling, we performed combinatorial intravitreal injections of anti-sFRP2 and Noggin. Once again, anti-sFRP2 or Noggin treatment alone resulted in increased EdU labeling in the CE relative to PBS control (Fig. 1P). Anti-sFRP2 and Noggin combined had a greater effect than Noggin alone, but not anti-sFRP2 alone, suggesting there is not an additive effect of antagonism of BMP proteins with sFRP2 antagonism. Once again, no effect on EdU labeling was observed in the NR, suggesting that sFRP2 and BMP blockade have specific effects in the CE.

We observed that some EdU^+^ cells in the CE and NR co-labelled for endothelial cell and microglia/marcrophage markers (Additional file 1: Figure S2, S3). As there are currently no markers that uniquely identify RSCs in vivo, we sought to refine our EdU labeling analysis to specifically analyze CE cells. Pax6 labels both layers of the CE, is a marker of retinal progenitors, and is highly expressed and functionally required in RSCs [42–44]. Henceforth, we quantified the number of EdU^+^ cells that were co-labeled with Pax6 to determine the level of CE-specific proliferation. Anti-sFRP2 and Noggin interfere with sFRP2 and BMP ligand-receptor interactions at the extracellular level, but they are expected to mediate their effects by modulating downstream Wnt and BMP signaling, respectively. To investigate the effects of modulating downstream Wnt and BMP signaling more directly, we injected adult mouse eyes with two ATP-competitive small molecule inhibitors that act at the intracellular level (Additional file 1: Figure S4A). GSK3β inhibitor CHIR99021 disrupts the β-catenin destruction complex, enabling β-catenin to enter the nucleus and activate canonical Wnt target genes [28]. BMP inhibitor LDN-193189 selectively inhibits BMP receptor kinases, preventing SMAD phosphorylation and translocation to BMP target genes [45]. Using Pax6 EdU analysis, we found that CHIR99021 and LDN-193189 each significantly increased the number of Pax6^+^EdU^+^ CE cells relative to DMSO control (Additional file 1: Figure S4C). These findings confirm that modulating the BMP and Wnt signaling pathways can induce CE cell proliferation.

RSCs can divide symmetrically to generate two stem cells and increase RSC number, or divide asymmetrically to generate one stem cell and one progenitor cell and maintain the RSC pool [1, 2, 17, 46]. In vitro*,* single RSCs proliferate to form clonal spheres of cells. Thus, the number of RSC spheres is a measure of the number of endogenous RSCs and can be used to detect changes in mode of division [1, 46]. To determine if injection of anti-sFRP2 or Noggin modulates the numbers of RSCs in vivo, we injected Noggin or anti-sFRP2 followed by a clonal sphere-forming assay*.* Seven days following the intravitreal injection period, the CE was dissected, dissociated to single cells, then plated at clonal density (10 cells/µL) for a 7-day sphere forming assay (Fig. 1Q). Exploratory dose–response experiments were used to identify concentrations of the injected factors that appeared to increase sphere number, which were then used in subsequent experiments. Anti-sFRP2 resulted in a significant increase in RSC sphere number, with a ~ 2.2-fold increase relative to PBS control (Fig. 1R). Noggin resulted in a significant increase in RSC sphere number, with a ~ 2.4-fold increase relative to PBS control (Fig. 1S). Thus, intravitreal injection of anti-sFRP2 or Noggin expands the endogenous RSC population. Despite the increases in sphere number, no change in sphere diameter was observed after injection of either factor. All together, these results demonstrate that antagonism of sFRP2 or BMP proteins in the adult mouse vitreous induces CE-specific proliferation and can expand the RSC pool in vivo.

### Noggin, anti-sFRP2, FGF2 and Insulin have differential effects on CE proliferation versus retinal stem cell expansion

**Fig. 2:**
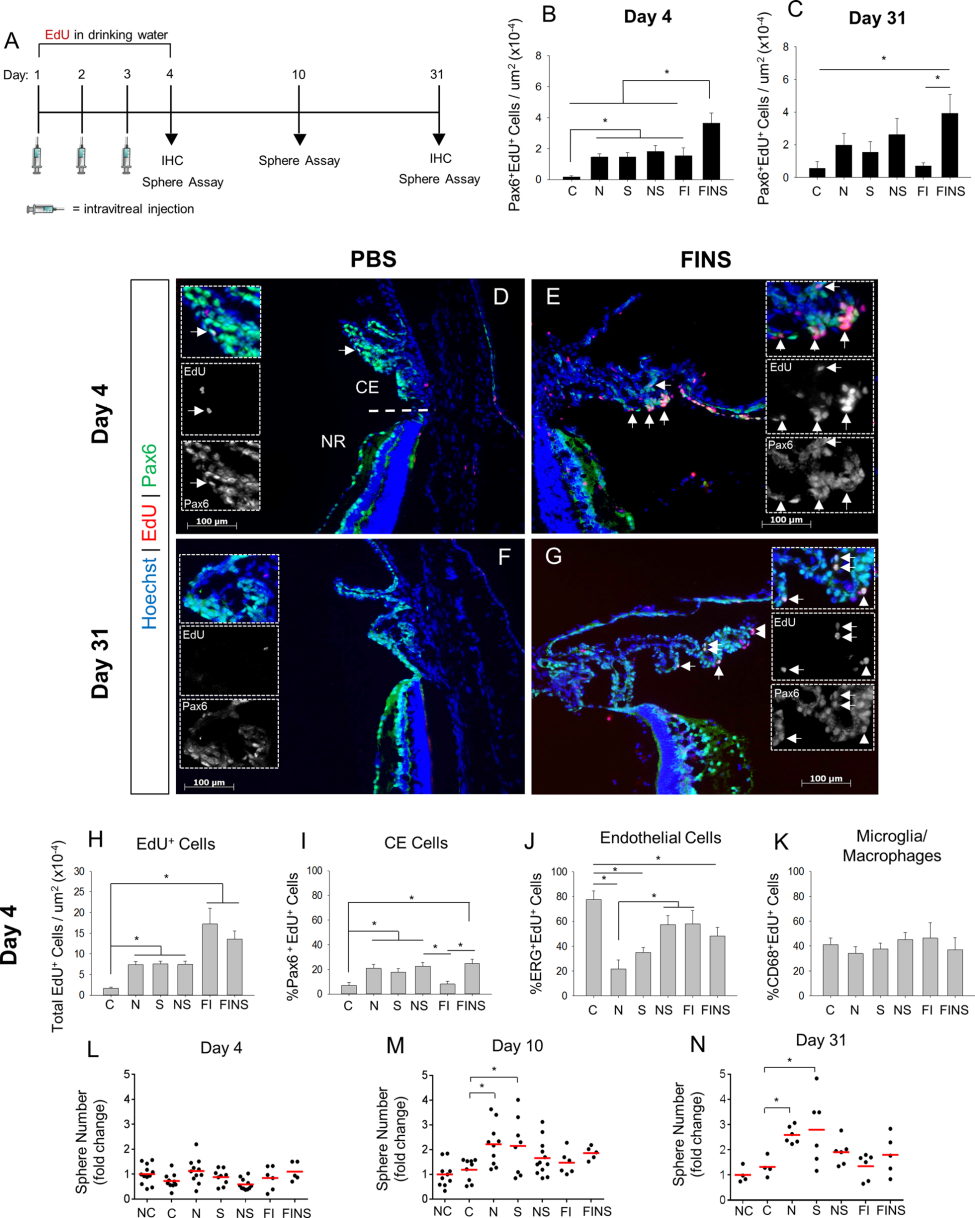
Combinatorial injection of Noggin and anti-sFRP2 with and without growth factors have differential effects on CE proliferation and retinal stem cell expansion. **A** Schematic of the intravitreal injection paradigm followed by endpoint clonal retinal stem cell (RSC) sphere forming assay from primary ciliary epithelium (CE) and/or immunohistochemistry (IHC). Mice received one intravitreal injection per day for three days followed by endpoint analysis at Day 4, 10 or 31. Injections consisted of: C = PBS control, N = Noggin, S = anti-sFRP2, NS = Noggin + anti-sFRP2 combined, FI = FGF2 + Insulin combined, FINS = FGF2 + Insulin + Noggin + anti-sFRP2 combined. **B, C** Quantification of Pax6^+^EdU^+^ co-labeled cells relative to total CE area in eyes treated with PBS vehicle or indicated factors. **B** Day 4 Pax6^+^EdU^+^ cells. (*p* < 0.001; N = 5–6 eyes per group). **c** Day 4 Pax6^+^EdU^+^ cells. (*p* = 0.037; N = 3–6 eyes per group). **D-G** Pax6 immunostaining and EdU labeling in the ciliary epithelium and peripheral retina of eyes injected with PBS vehicle or indicated factors at Day 4 and Day 31. Hoechst stain was used to label all nuclei. White arrows indicate Pax6^+^EdU^+^ double-positive cells. Straight dashed line indicates the border between the ciliary epithelium (CE) and the neural retina (NR). Dashed line box indicates high magnification inset. **H** Quantification of EdU cell number in the CE normalized by area in eyes treated with the indicated conditions. (*p* < 0.001; N = 5–6 eyes per group). **I-K** Percent of total EdU-positive cells in the CE that co-labeled for cell-type-specific markers in eyes treated with the indicated conditions. **i** % Pax6^+^EdU^+^ CE cells. (*p* < 0.001; N = 5–6 eyes per group). **J** % ERG^+^EdU^+^ endothelial cells. (*p* < 0.001; N = 5–6 eyes per group). **K** % CD68^+^EdU^+^ microglia/macrophage cells. N = 5–6 eyes per group. **L-N** Quantification of RSC sphere frequency normalized to naïve un-injected control following intravitreal injection of the indicated factors. **L** Day 4. N = 5–12 eyes per group. **M** Day 10. (*p* = 0.003; N = 5–12 eyes per group). **N** Day 31. (*p* = 0.001; N = 4–6 eyes per group). Each data point represents a single eye. Statistics: One-way ANOVAs with Holm-Sidak posthoc tests. Data are means ± SEMs. * = *p* < 0.05

Given the evidence that BMP and sFRP2 are negative regulators of RSC proliferation in vivo, we hypothesized that antagonism of BMPs and sFRP2 may render RSCs more amenable to growth factor stimulation. We chose FGF2 and Insulin as our growth factors of interest due to the importance of FGF and Insulin/IGF signaling in retinogenesis [47], evidence that FGF is imperative for adult RSC proliferation in vitro (Balenci and van der Kooy, 2014; Tropepe et al., 2000), and because FGF2 and Insulin have been reported to stimulate CE proliferation and neurogenesis in the perinatal CE/CMZ [48, 49]. We examined two outcome measures in this experiment: 1) immunohistochemistry (IHC) to determine the number of Pax6^+^EdU^+^ co-labeled CE cells; and 2) a clonal, sphere-forming assay as a proxy for the number of RSCs in vivo (Fig. 2A). Intravitreal injections included 6 different conditions: PBS control (C), Noggin (N), anti-sFRP2 (S), Noggin + anti-SFRP2 (NS), FGF2 + Insulin (FI) and all factors combined (FINS). The primary sphere assays were normalized to naïve un-injected controls (NC). At Day 4, the number of Pax6^+^EdU^+^ CE cells was greater in all treatment conditions relative to PBS control (Fig. 2B, Additional file 1: Figure S5). FI alone increased CE proliferation to a similar extent as Noggin and anti-sFRP2 combined, revealing that growth factors can stimulate CE cell proliferation even with the RSC quiescence factors present. However, the largest effect was observed in the FINS treated eyes, which resulted in a ~ 21-fold increase in Pax6^+^EdU^+^ cells compared to PBS, and a ~ twofold increase compared to NS, which had the second highest effect. FINS also resulted in the largest clusters of Pax6 + EdU^+^ cells evident in eye sections (Fig. 2E;Additional file 1: Figure S5F). Thus, sFRP2 and BMP antagonism and growth factor stimulation have an additive effect on CE proliferation. At Day 31, only the FINS group retained a significantly greater number of Pax6^+^EdU^+^ cells relative to PBS control (Fig. 2C,G; Additional file 1: Figure S6).

The ciliary processes are richly endowed with blood capillaries and resident tissue macrophages [7, 50]. Thus, we hypothesized that endothelial cells and marcophages are potential “off-target”, non-CE, cell types that can incorporate EdU and potentially confound EdU label analysis in the tissue. In order to determine to what extent these non-CE cell types are stimulated to proliferate, we quantified the proportion of total EdU^+^ cells in the CE that were co-labeled with Pax6 (CE cells), ERG (a nuclear marker for endothelial cells), or CD68 (a marker of microglia/macrophages). All conditions significantly increased total EdU labeling in the CE compared to PBS control, but FI and FINS had roughly twice the effect of the other treatment groups (Fig. 2H). Despite a similar total number of EdU^+^ cells in the CE after FI and FINS treatment, only ~ 8% of EdU^+^ cells co-labeled with Pax6 in FI treated eyes, similar to PBS control (~ 7%) (Fig. 2I). In contrast, FINS resulted in the highest proportion of EdU^+^Pax6^+^ cells at ~ 25%. However, FINS induced proportionally more ERG^+^ endothelial cell proliferation (48%) than Noggin or anti-sFRP2 (22% and 35%, respectively) (Fig. 2J). Yet, all three of those conditions had a lower proportion of EdU^+^ERG^+^ cells than PBS control (80%). In addition, a significant proportion of EdU + cells co-labeled for the microglia/macrophage marker CD68 in all conditions (Fig. 2K). However, all conditions were proportionally the same, indicating that none of the injected factors increased microglia activation or macrophage infiltration. Thus, antagonism of sFRP2 or BMP proteins induces proliferation of CE cells and enhances the potency and specificity of growth factor stimulation of CE cells. Also notable, we did not observe EdU^+^ cells in the RPE layer, nor any Sox9 (Müller glia marker) and EdU co-labeled cells in the neural retina, suggesting that RPE and Müller glia do not proliferate in response to the injected factors.

Next, we performed combinatorial intravitreal injections of Noggin, anti-sFRP2 and growth factors, followed by clonal sphere-forming assays to assess the effects on the number of RSCs in vivo (Fig. 2A). At Day 4, no treatment condition had any effect on RSC sphere number (Fig. 2L). At Day 10, only anti-sFRP2 or Noggin alone increased the number of RSC spheres (Fig. 2M). Surprisingly, the combined NS condition did not have a significant effect at the Day 10 timepoint, nor did FI, or all factors combined (FINS). At Day 31, once again, only individual anti-sFRP2 or Noggin treatments resulted in increased RSC sphere number (Fig. 2N). Therefore, although sFRP2 and BMP antagonism combined with growth factor stimulation resulted in the largest stimulation of CE cell proliferation, it did not result in expansion of the RSC pool. Thus, if CE proliferation is indicative of RSC division, RSCs appear to divide asymmetrically under most conditions, while symmetric expansion of the RSC population occurs only with discrete antagonism of BMPs or sFRP2. In addition, all symmetric expansion appeared to occur between Day 4 and Day 10. Thus, the earliest cell divisions, when RSCs are exiting quiescence, appear to be asymmetric divisions. Furthermore, the expanded endogenous RSC pool persists up to one month later but does not continue to expand beyond the extent observed at Day 10.

### Inducible Msx1-Cre^ERT2^ lineage labeling marks the adult ciliary epithelium and retinal stem cells

**Fig. 3:**
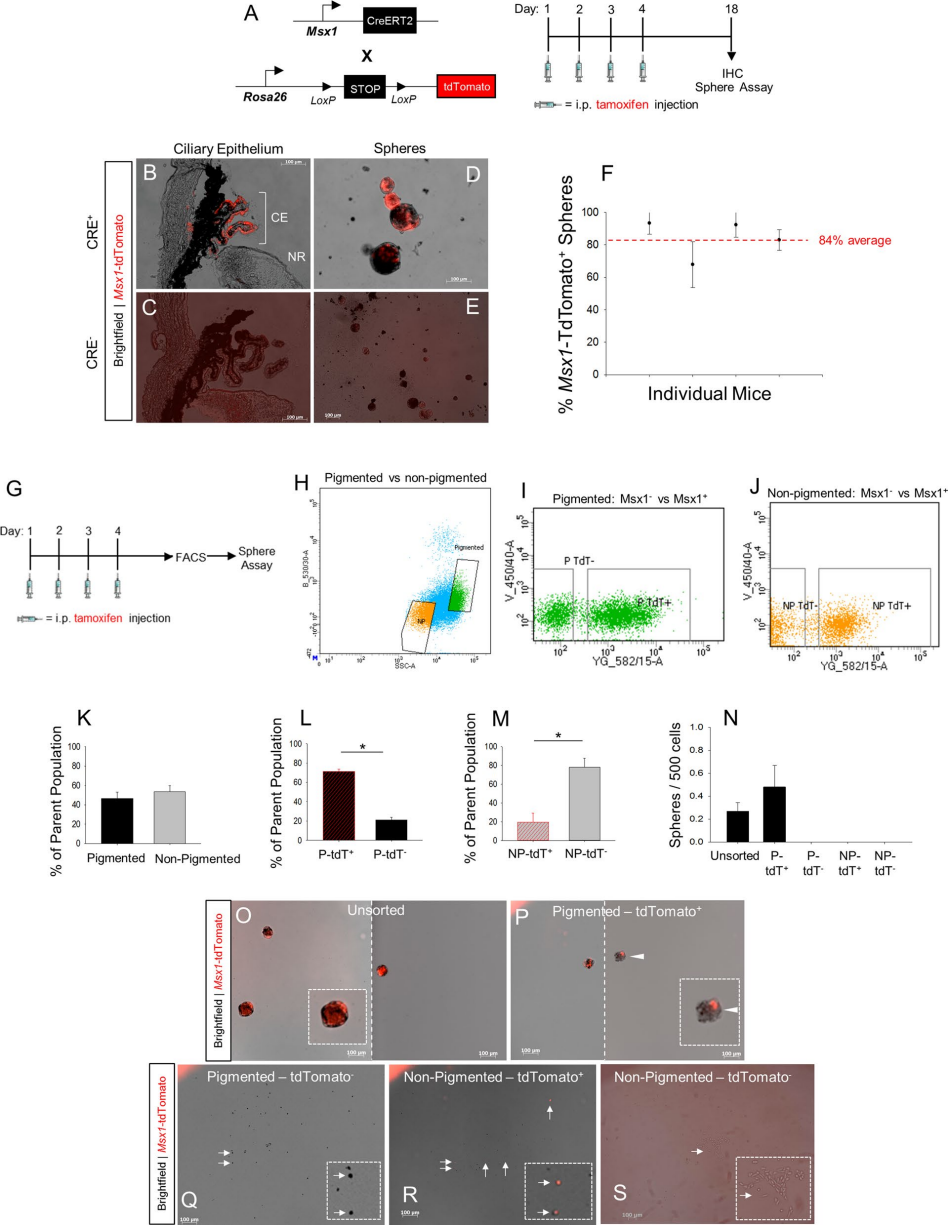
Tamoxifen induction of reporter expression in Msx1-Cre^ERT2^;Rosa26-tdTomato mice labels the ciliary epithelium and CE-derived RSC spheres. **A** Schematic of Msx1-Cre^ERT2^;Rosa26-tdTomato mouse lines and transgene induction paradigm. Four consecutive daily intraperitoneal tamoxifen injections were followed by endpoint clonal RSC sphere assay and immunohistochemistry (IHC) two weeks after the injection period. **B-E** Fluorescence microscopy of eye sections and RSC spheres from the eyes of Msx1-Cre^ERT2^;Rosa26-tdTomato mice two weeks post-tamoxifen injection. **B-D** Msx1-Cre^+^ mice. **C-E** Littermate control Msx1-Cre^−^mice. A lower magnification image is shown compared to panel D to demonstrate that there were no positive spheres in a broad field of view. CE = ciliary epithelium, NR = neural retina. **F** The Percentage of RSC spheres with *Msx1*-tdTomato expression derived from tamoxifen-treated Msx1-Cre^ERT2^;Rosa26-tdTomato mice. Each data point represents the average % labeled RSC spheres per well for a single mouse. Red dashed line indicates between mouse average. N = 4 mice. **G** Schematic of Msx1-Cre^ERT2^;Rosa26-tdTomato transgene induction followed by fluorescence activated cell sorting (FACS) and subsequent clonal RSC sphere assay. **H-J** Representative FACS gating plots for CE cells derived from Msx1-Cre^ERT2^;Rosa26-tdTomato mice following tdTomato reporter induction. N* p* = non-pigmented; *p* = pigmented; tdT +  = tdTomato positive; tdT- = tdTomato negative. **K-M** The proportions of each FACS subpopulation. **K** The proportion of pigmented and non-pigmented cells. **L** The proportion of *Msx1*-tdTomato^+^ and *Msx1*-tdTomato^−^ cells within the pigmented CE population. (*p* < 0.001; N = 4 FACS experiments). **M** The proportion of *Msx1*-tdTomato^+^ and *Msx1*-tdTomato^−^ cells within the non-pigmented CE population. (*p* = 0.005; N = 4 FACS experiments). **N** The number of RSC spheres that formed 7 days after sorting for each FACS subpopulation. N = 3 FACS experiments. **o-s** Representative images of RSC sphere assay output wells for each sorted subpopulation 7 days following FACS. **O** Unsorted CE cells. Two images stitched together (indicated by the dashed line). **P** Pigmented *Msx1*-tdTomato^+^ CE cells. Two images stitched together (indicated by the dashed line). The white arrowhead indicates a sphere that has partial expression of tdTomato. **Q** Pigmented *Msx1*-tdTomato^−^ CE cells. **R** Non-pigmented *Msx1*-tdTomato^+^ CE cells. **S** Non-pigmented *Msx1*-tdTomato^−^ CE cells. White arrows indicate single *Msx1*-tdTomato expressing cells. Dashed line box indicates high magnification inset. Statistics: t-tests. Data are means ± SEMs. * = *p* < 0.05

To assess CE cell migration or neurogenesis by profiling EdU^+^ cells in the retina is not conclusive, given that some EdU^+^ cells are detectable in the retina immediately following intravitreal injection. Thus, we sought to determine if an in vivo genetic lineage tracing model could be used to label and track CE cells in adult mouse eyes. We used the previously generated tamoxifen-inducible *Msx1-Cre*^*ERT2*^ knock-in mouse line [36] and crossed it with the B6.Cg*-Gt(Rosa26)Sor*^*Tm14(CAG−tdTomato)Hze*^ tdTomato reporter mouse line [35] (Fig. 3A).

To determine the specificity of reporter expression, *Cre*^+^ and *Cre*^*−*^* Msx1-Cre*^*ERT2*^*;Rosa-tdTomato* littermate adult mice were injected i.p. with tamoxifen (180 mg/kg) for 4 consecutive days (Fig. 3A). *Cre*^+^ mice had *Msx1*-tdTomato reporter expression in the entire (proximal and distal) CE (Fig. 3B), although there were varying degrees of penetrance. Regardless of the degree of penetrance, *Msx1*-tdTomato labeling consistently overlapped with Pax6 labeling in the CE (Fig S7). In contrast, *Cre*^*−*^ mice did not show any *Msx1*-tdTomato reporter labeling in the CE (Fig. 3C). Additionally, we found that RSC spheres derived from *Cre*^+^ mice expressed the *Msx1*-tdTomato reporter (Fig. 3D). As single, *Msx1*-tdTomato^+^ RSCs would be required for tdTomato^+^ clonal RSC spheres to arise, this is the first evidence we are aware of that adult RSCs express *Msx1*. Further, roughly 84% of all RSC spheres had *Msx1*-tdTomato^+^ cells indicating the majority of RSCs are labeled (Fig. 3F). The clonal spheres from *Cre*^+^ mice without *Msx1*-tdTomato labeling may have arisen due to variable efficiency of tamoxifen induction. None of the RSC spheres derived from the eyes of *Cre*^*−*^ mice were labeled with *Msx1*-tdTomato (Fig. 3E). In order to further characterize reporter expression within the CE, we performed FACS on primary CE cells from mice that had received tamoxifen induction 1–2 weeks prior (Fig. 3G). The CE was sorted into 4 populations of cells: pigmented tdTomato-negative cells (P-tdT^−^), pigmented tdTomato-positive cells (P-tdT^+^), non-pigmented tdTomato-negative cells (NP-tdT^−^), and non-pigmented tdTomato-positive cells (P-tdT^+^) (Fig. 3H-J). After sorting, each population was plated at 1 cell/µL for a 7-day clonal sphere-forming assay. As expected, pigmented and non-pigmented CE were nearly equivalent proportions of the CE (46.6% pigmented, 53.4% non-pigmented) (Fig. 3K). However, on average 71% of pigmented CE cells were *Msx1*-tdTomato^+^ (Fig. 3L), whereas only 20% of non-pigmented CE cells were *Msx1*-tdTomato^+^ (Fig. 3M). The post-FACS sphere assay revealed that only P-tdT^+^ CE cells could generate RSC spheres (Fig. 3N-S). This is concordant with previous reports that sphere-forming RSCs are exclusive to pigmented cells in the outer CE [1, 5]. However, this suggests that RSCs may be exclusively *Msx1*-expressing pigmented CE cells. Although there was a trend toward increased sphere frequency in the P-tdT^+^ population relative to the unsorted control, sorting did not lead to a significant enrichment of RSCs (Fig. 3N). However, only ~ 25% live cells were recovered during FACS, therefore RSC sphere frequency after sorting likely underrepresents the actual RSC number (Additional file 1: Fig S8A-E). This may explain why no spheres arose in the P-tdT^−^ sorted population.

In some RSC spheres, not all cells were *Msx1*-tdTomato^+^. This was true for spheres formed using the regular sphere assay paradigm with non-sorted cells (Fig. 3D, bottom sphere), as well as spheres formed post-FACS assay (Fig. 3P, white arrowhead).To investigate if transgene silencing was occurring, we extracted DNA from single spheres for PCR analysis using primers spanning the floxed stop sequence of the *Rosa-tdTomato* reporter construct. In clonal spheres, a single band at 2 Kb (stop sequence present) or 1 Kb (stop sequence excised) is expected. If, however, there was non-clonal mixing of cells with both genotypes then both bands would be expected to appear in the PCR gel. Out of 21 spheres analyzed, only one had bands at both the 1 Kb and 2 Kb molecular weights. Thus, in 95% of spheres, all cells had a single genotype (Additional file 1: Figure S8F). Therefore, it is likely that spheres with incomplete reporter labeling had heterogeneous transgene silencing in a subset of cells in the clonal RSC spheres. Together, these results indicate that the *Msx1-Cre*^*ERT2*^ mouse line can be used to lineage label the adult mouse CE and reveals that adult RSCs and RSC-derived progenitors are labeled by this paradigm. However, we predict it will underrepresent the extent of CE cell migration and differentiation due to incomplete tamoxifen penetrance and transgene silencing.

### FINS-mediated CE proliferation is potentiated by photoreceptor degeneration

**Fig. 4:**
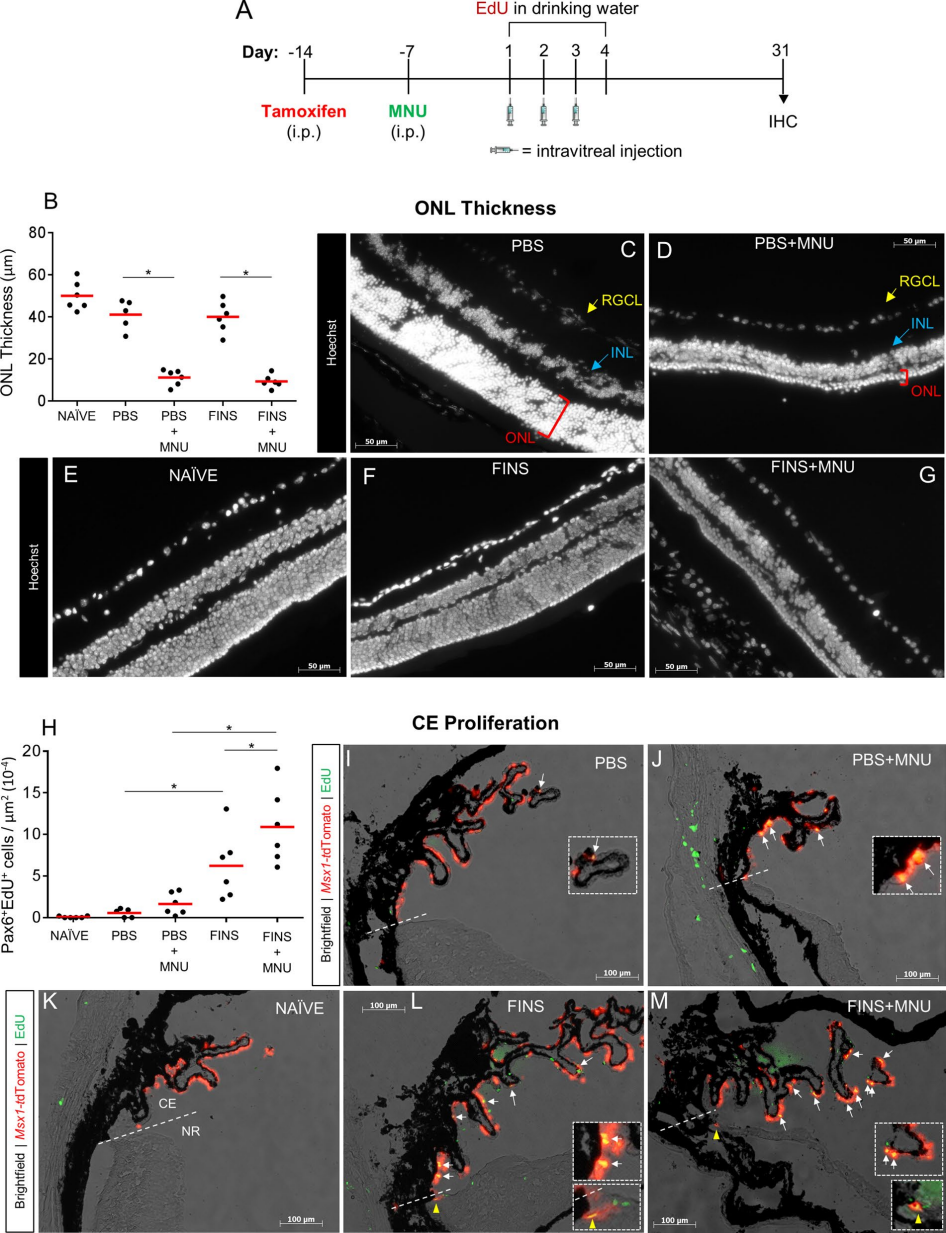
FINS-mediated CE proliferation is augmented by photoreceptor degeneration in Msx1-Cre^ERT2^;Rosa26-tdTomato mice. **A** Schematic of the lineage tracing paradigm in tamoxifen-treated Msx1-Cre^ERT2^;Rosa26-tdTomato mice used to investigate the effects of MNU injury and/or FINS (FGF2 + Insulin + Noggin + anti-sFRP2) treatment on ciliary epithelium proliferation, migration and differentiation. The injection period was followed by tissue fixation and IHC analysis at Day 31. Groups included: Naïve control = no injection; PBS = intravitreal PBS; PBS + MNU = intravitreal PBS and i.p. MNU; FINS = intravitreal FINS; FINS + MNU = intravitreal FINS and i.p. MNU. **B** Quantification of outer nuclear layer (ONL) thickness in eyes from mice of the indicated conditions. (*p* < 0.001; N = 6 eyes per group). Each data point represents a single eye. **C-G** Representative images of Hoechst nuclear-stained eye cross-sections from mice of the indicated conditions. RGCL = retinal ganglion cell layer; yellow indicators. INL = inner nuclear layer; blue indicators. ONL = outer nuclear layer; red indicators. **H** Quantification of Pax6^+^EdU^+^ co-labeled cells relative to total CE area in eyes from mice of the indicated conditions. There was a significant effect of treatment (*p* < 0.001) and a significant effect of MNU injury (*p* = 0.035). N = 6 eyes per group. Each data point represents a single eye. **I-M** Brightfield and fluorescence overlay images of *Msx1*-tdTomato expression and EdU labeling in eyes from mice of the indicated conditions. White arrows indicate *Msx1*-tdTomato^+^EdU^+^ co-labeled cells in the ciliary epithelium. Yellow arrowheads indicate *Msx1*-tdTomato^+^EdU^+^ co-labeled cells in the retina. Straight dashed line indicates the border between the ciliary epithelium (CE) and the neural retina (NR). Dashed line box indicates high magnification inset. Statistics: Two-way ANOVAs with Holm-Sidak posthoc tests except where stated otherwise. Means ± SEMs indicated. * = *p* < 0.05

Thus far, our analyses of CE proliferation had been in mice with healthy retinas (with the caveat that intravitreal injection results in minor retinal damage). Therefore, in addition to examining the effects of FINS treatment, we used the *Msx1-Cre*^*ERT2*^ mouse line to assess if retinal injury would influence CE proliferation, migration or differentiation. To induce photoreceptor degeneration, we used the *N*-methyl-*N*-nitrosourea (MNU) injury model (Fig. 4A). MNU is a DNA alkylating agent that induces photoreceptor-specific degeneration via multiple cell death pathways [51, 52] that is apparent as a reduction in outer nuclear layer (ONL) thickness (Figure S9). A naïve un-injected group was used to control for the effects of intravitreal injection. Therefore, 5 groups were included in this experiment: Naïve control, PBS, PBS + MNU, FINS and FINS + MNU. All mice received EdU in their drinking water during the injection period. Some PBS + MNU eyes had pathology indicative of phthisis bulbi and were excluded from further analyses (Figure S10). Otherwise, no difference in ONL thickness between PBS + MNU mice and FINS + MNU mice was observed (Fig. 4B). Both PBS + MNU and FINS + MNU eyes had significantly reduced ONL thickness compared to the un-injured groups, indicating FINS treatment did not ameliorate photoreceptor degeneration at this gross level (Fig. 4B-G). Due to the incomplete penetrance of *Msx1*-tdTomato expression in the CE, we again used Pax6 to quantify EdU labeling in the CE (Fig. 4H). PBS + MNU did not significantly increase proliferation compared to PBS alone. Yet, there was a pronounced increase in the effect of FINS injection in MNU injured eyes. Indeed, the FINS + MNU group had the highest level of Pax6^+^EdU^+^ labeling in the CE of all conditions tested in this study, suggesting the CE had a greater proliferative response to FINS when injury was present. Co-labeled EdU^+^*Msx1*-tdTomato^+^ CE cells were readily apparent, and the extent of visible EdU labeling correlated well with the Pax6^+^EdU^+^ cell quantification across conditions (Fig. 4I-M; Figure S11). Furthermore, EdU^+^*Msx1*-tdTomato^+^ cells were apparent in the retina, suggesting at least some of the EdU^+^ cells observed in the retina are derived from the CE (yellow arrowheads). Thus, *Msx1*-tdTomato served as a second CE-specific marker to validate that CE cells proliferate in response to FINS and provided evidence that some proliferating CE cells may migrate into the retina. Also, these results revealed that MNU injury potentiates FINS stimulation of CE proliferation.

### Photoreceptor degeneration and FINS treatment induce CE cell migration into the neural retina

In order to qualify what constitutes CE migration into the peripheral neural retina, we analyzed the domain of *Msx1*-mediated tdTomato expression in naïve mouse eyes 1 day and 45 days after the tamoxifen injection paradigm (Additional file 1: Figure S12A-E). The farthest into the retina an *Msx1*-tdTomato^+^ cell was detected at Day 1 was 10 µm. Therefore, an *Msx1*-tdTomato^+^ cell needed to be beyond 10 µm into the retina to be included in the experimental migration analyses below (Additional file 1: Figure S12B). Of note, only 7 cells total were observed beyond 10 µm in Day 45 naïve eyes, after analyzing a combined total of 120 sections from 6 eyes (20 sections per eye). However, that represented a significant increase in migration distance at Day 45 compared to Day 1 (Additional file 1: Figure S12C). This suggests that, even in naïve eyes, there may be cell migration into the retina at extremely low frequency.

**Fig. 5:**
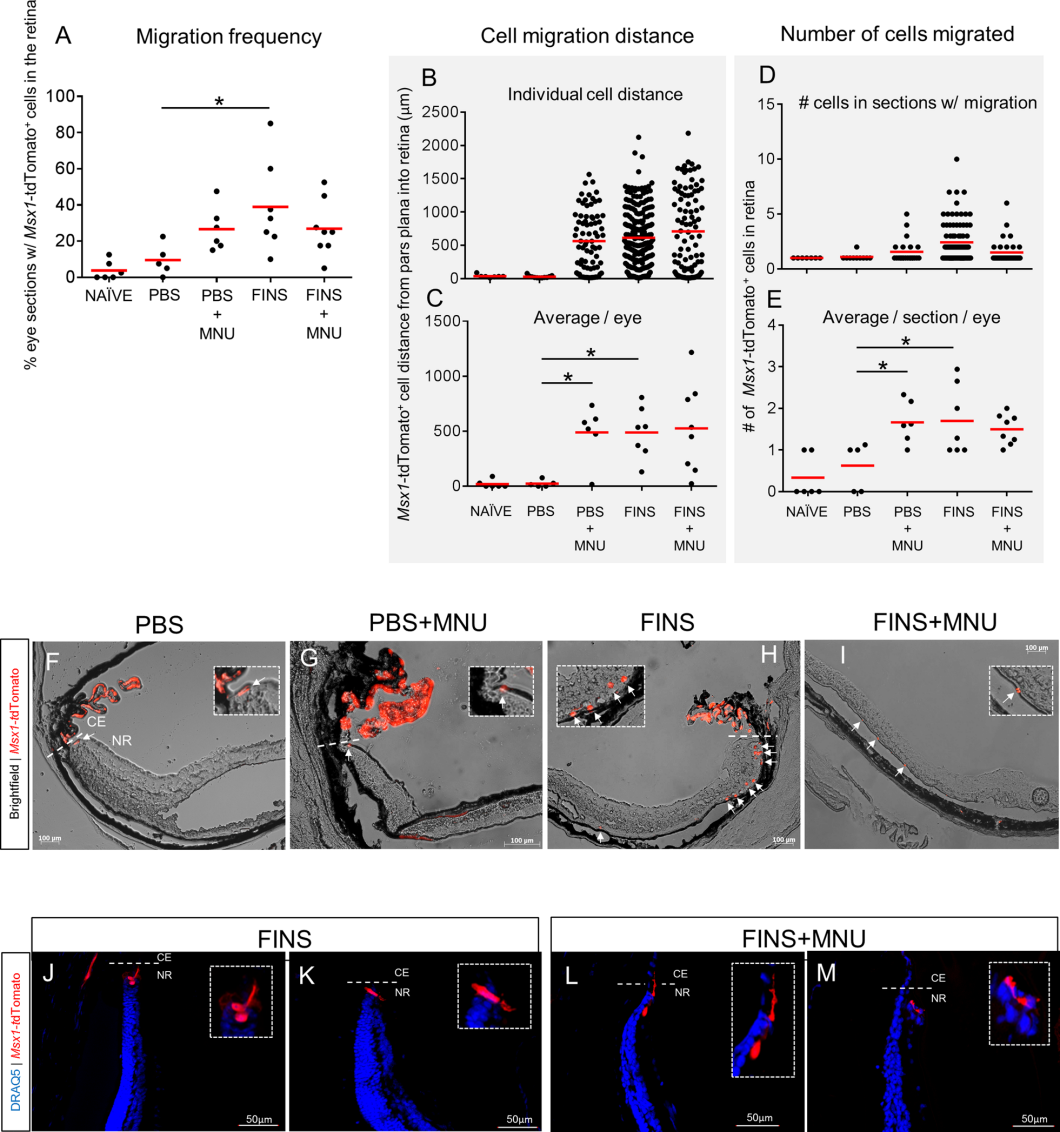
Photoreceptor degeneration or FINS treatment induces ciliary epithelial cell migration into the neural retina. **A-I** Analyses of eye slides from the endpoint of the experimental paradigm outlined in Fig. 4A (20 sections analyzed per eye). **A** Percent of eye sections with *Msx1*-tdTomato^+^ cells present in retina for mice of the indicated conditions. There was a significant interaction between injury and treatment on migration frequency (*p* = 0.021; N = 6–8 eyes per group). Each data point represents a single eye. **B, C** Quantification of the migration distance of *Msx1*-tdTomato^+^ cells into the retina for mice of the indicated conditions. **B** The migration distance recorded for individual cells. Each data point represents a single cell, N = 9–228 cells from 6–8 eyes per group. **c** Average migration distance per eye. There was a significant effect of treatment (*p* = 0.029) and injury (*p* = 0.028). N = 6–8 eyes per group. Each data point represents a single eye. **D-E** Quantification of the number of *Msx1*-tdTomato^+^ cells in the retina for mice of the indicated conditions. **D** The total number of cells recorded in individual retina sections. Each data point represents a single retina section with at least one *Msx1*-tdTomato^+^ cell detected. **e** Average number of *Msx1*-tdTomato^+^ cells in retina sections per eye. There was a significant interaction between injury and treatment on the number of *Msx1*-tdTomato^+^ cells in the retina (*p* = 0.007; N = 6–8 eyes per group). Each data point represents a single eye. **F-I** Brightfield and fluorescence overlay images of *Msx1*-tdTomato^+^ cells in retinas from mice treated with the indicated conditions. White arrows indicate *Msx1*-tdTomato^+^ cells in the retina. **J-M** Msx1-tdTomato^+^ CE cells in the retina with neuroepithelial or neurite morphology at Day 31. DRAQ5 stain was used to label all nuclei. Straight dashed line indicates the border between the ciliary epithelium (CE) and the neural retina (NR). Dashed line box indicates high magnification inset. Statistics: Two-way ANOVAs with Holm-Sidak posthoc tests. Means ± SEMs indicated. * = *p* < 0.05

PBS injection alone did not increase the frequency of cell migration, cell migration distance, or the number of cells that migrated per section compared to naïve eyes (Fig. 5A-E). FINS injection alone significantly increased migration frequency, with an average of ~ 39% of eye sections showing CE cell migration per eye (Fig. 5A). However, unlike proliferation, there was not an increased migration effect in the FINS + MNU injury group compared to FINS alone. Although there appeared to be a trend toward increased migration frequency in the PBS + MNU group, it was not statistically different from PBS alone. In contrast, cell migration distance was markedly increased by both MNU injury and FINS treatment, as average migration distance in PBS eyes was ~ 20 µm, while PBS + MNU, FINS and FINS + MNU all averaged around 500 µm (Fig. 5B-C). Likewise, PBS + MNU, FINS and FINS + MNU all had similar cell number averages of ~ 1.5–1.8 cells per section (Fig. 5D-E). Both, the increased migration distance and cell number were evident in retinal sections (Fig. 5F-I). Panels 5H and 5I are examples of higher numbers of *Msx1*-tdTomato^+^ cells having migrated into the retina than the average indicated in 5E. Also, some *Msx1*-tdTomato^+^ cells in the retina had neuroepithelium-like morphology while others appeared to extend processes (Figue 5 J-M). These results reveal that photoreceptor degeneration and FINS stimulation can each induce CE migration into the retina, but that FINS stimulation has a greater effect.

### Msx1-tdTomato^+^ cells in the neural retina express photoreceptor or retinal ganglion cell markers

**Fig. 6:**
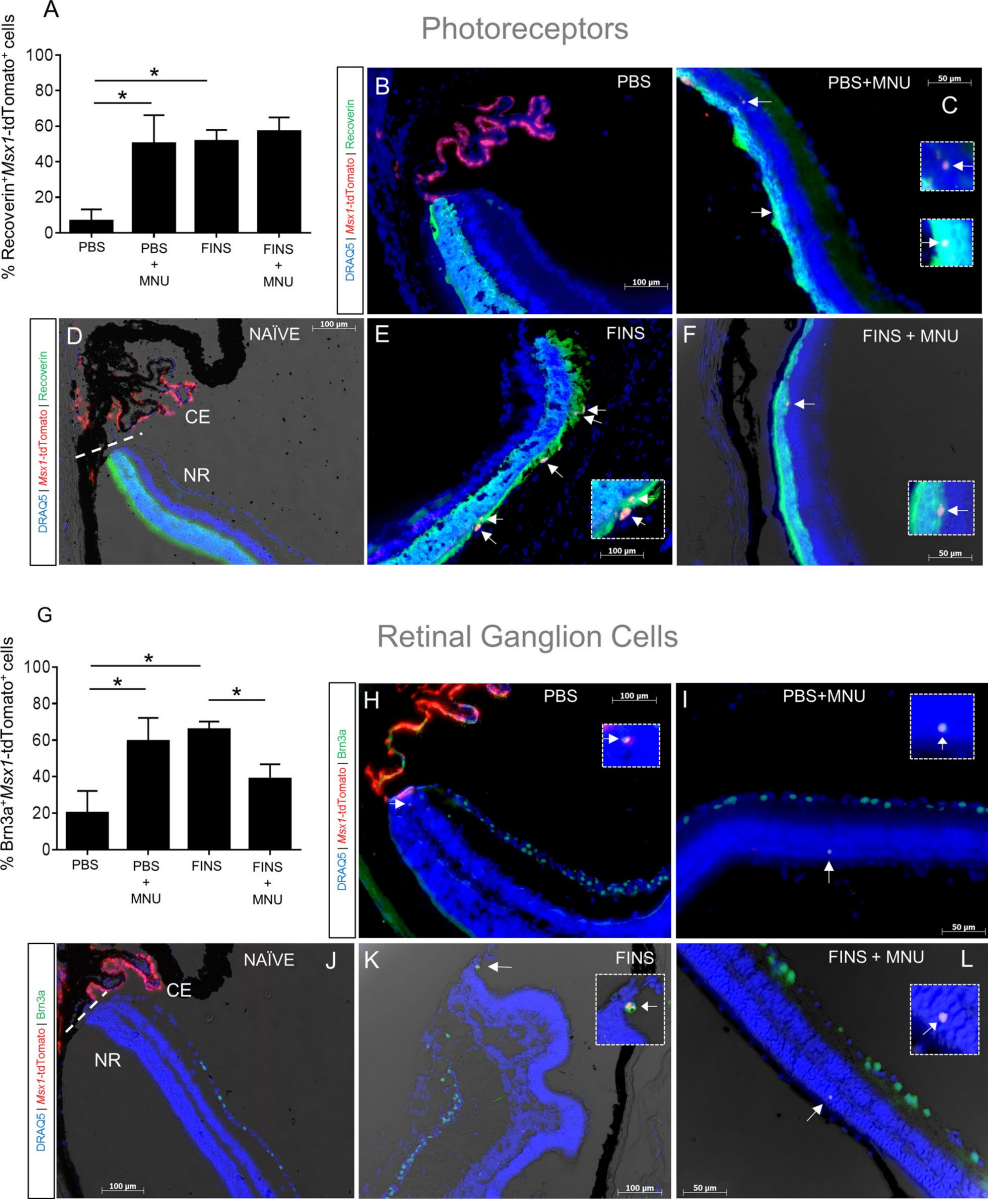
Ciliary epithelium derived tdTomato^+^ cells in the neural retina express photoreceptor or retinal ganglion cell markers. **A-L** Analyses of retinal cell type markers in eye slides from the endpoint of the experimental paradigm outlined in Fig. 4A**. A** Quantification of the percentage of *Msx1*-tdTomato^+^ cells in the retina that co-labeled for the photoreceptor marker Recoverin in eye sections from the indicated conditions. There was a significant effect of treatment (*p* = 0.026) and injury (*p* = 0.034) on the proportion of *Msx1*-tdTomato^+^Recoverin^+^ cells. N = 5–8 eyes per group, 3–6 slides per eye. **B-F** Brightfield and fluorescence overlay images of Msx1-Cre driven tdTomato expression and immunostaining for photoreceptor marker Recoverin in eye sections from the indicated conditions. DRAQ5 stain was used to label all nuclei. White arrows indicate *Msx1*-tdTomato^+^Recoverin^+^ co-labeled cells. **G** Quantification of the percentage of *Msx1*-tdTomato^+^ cells in the retina that co-labeled for the retinal ganglion cell marker Brn3a in eye sections from the indicated conditions. There was a significant interaction between treatment and injury on the proportion of *Msx1*-tdTomato^+^Brn3a^+^ cells (*p* = 0.002; N = 5–8 eyes per group, 3–6 slides per eye). **H–L** Brightfield and fluorescence overlay images of Msx1-Cre driven tdTomato expression and immunostaining for retinal ganglion cell marker Brn3a in eye sections from the indicated conditions. DRAQ5 stain was used to label all nuclei. White arrows indicate *Msx1*-tdTomato^+^Brn3a^+^ co-labeled cells. Straight dashed line indicates the border between the ciliary epithelium (CE) and the neural retina (NR). Dashed line box indicates high magnification inset. Statistics: Two-way ANOVAs with Holm-Sidak posthoc tests. Data are means ± SEMs.* = *p* < 0.05

Next, we examined if the *Msx1*-tdTomato^+^ CE cells that migrated into the retina showed evidence of differentiation into retinal neurons by co-expressing retinal cell type markers. In the MNU injury model, photoreceptors are selectively degenerated. Furthermore, using *Msx1-Cre*^*ERT2*^ lineage tracing, Belanger et al. (2016) reported that photoreceptors were the most abundant cell type produced by the embryonic CE during retinogenesis. Therefore, we examined whether *Msx1*-tdTomato^+^ cells in the retina co-expressed the mature photoreceptor marker Recoverin. We were not able to locate enough *Msx1*-tdTomato^+^ cells in the Naïve control sections to quantify co-expression for Recoverin. All other conditions exhibited *Msx1*-tdTomato^+^Recoverin^+^ co-labeled cells in the retina (Fig. 6A-F; Additional file 1: Figure S13). Some sections had *Msx1*-tdTomato^+^ cells with no co-labeling for recoverin (0%), some sections had a fraction of *Msx1*-tdTomato^+^ cells with co-labeling, while other sections contained one or more *Msx1*-tdTomato^+^ cells that were all co-labeled (100%). For each eye, the total number of *Msx1*-tdTomato^+^recoverin^+^ cells relative to total *Msx1*-tdTomato^+^ cells was used to calculate co-expression percentages (Fig. 6A). Both MNU injury and FINS treatment resulted in a greater proportion (~ 50–55%) of *Msx1*-tdTomato^+^ cells in the retina that co-labeled for Recoverin relative to the PBS control (~ 7%) (Fig. 6A). In fluorescence images, *Msx1*-tdTomato^+^Recoverin^+^ cells were usually located apposed to the ONL between the ONL and RPE (Fig. 6C,E; Additional file 1: Figure S13C,D) or embedded within the ONL (Fig. 6F; Additional file 1: Figure S13E). Of note, the *Msx1*-tdTomato^+^Recoverin^+^ cells in the retina did not appear to have mature rod or cone photoreceptor morphology with inner and outer segments.

A study by Marcucci et al. (2016) reported that a subpopulation of proliferative, Cyclin D2^+^ embryonic CE cells migrate into the retina and generate retinal ganglion cells (RGCs) during retinogenesis. Therefore, we examined whether *Msx1*-tdTomato^+^ cells in the retina co-expressed the RGC marker Brn3a. Once again, we were not able to locate enough *Msx1*-tdTomato^+^ cells in the Naïve control sections to quantify co-expression for Brn3a. In PBS injected eyes, ~ 20% of *Msx1*-tdTomato^+^ cells detectable in the retina co-labeled for Brn3a (Fig. 6G). In both PBS + MNU and FINS eyes there was a greater proportion of *Msx1*-tdTomato^+^Brn3a^+^ co-labeled cells in the retina relative to PBS (60% and 65%, respectively). However, there was a smaller proportion of *Msx1*-tdTomato^+^ cells that co-labeled for Brn3a^+^ in the retinas of FINS + MNU mice than in FINS mice. In fluorescence images (Fig. 6H-L; Additional file 1: Figure S14), *Msx1*-tdTomato^+^Brn3a^+^ cells were usually located adjacent to the CE in the peripheral retina (Fig. 6H,K; Figure S14B,D) or embedded within the ONL (Fig. 6I,L; Additional file 1: Figure S13C,E). Of note, *Msx1*-tdTomato^+^Brn3a^+^ co-labeled cells did not extend neurites and were not observed in the RGC layer. However, in general, *Msx1*-tdTomato^+^ cells were rarely detected in the RGC layer in our migration analyses. Also, though extremely infrequent, some *Msx1-*tdTomato^+^ cells were detected in the RPE layer (Additional file 1: Figure S15).

## Discussion

In this study we found that intravitreal delivery of BMP antagonist, Noggin, or a function blocking antibody against sFRP2, can each induce proliferation of the adult CE in vivo. This is consistent with the evidence that endogenous BMP and sFRP2 proteins regulate the proliferation of RSCs in vitro [23]. We also found that antagonism of BMP and sFRP2 proteins made the RSC niche more responsive to stimulation with exogenous growth factors. Previous attempts to reactivate RSCs in the adult mammalian eye with exogenous factors have resulted in reports of some limited proliferation in the CE [53–55]. However, those studies did not use CE cell type markers to quantify proliferation, and instead, included all proliferating cells in the region in their analyses. Also, some of those studies evaluated nestin expression as a putative retinal stem cell marker, however nestin also marks endothelial cells [56] and microglia [57] in the retina and CE. Thus, it is unknown to what extent proliferation in non-CE cells, such as endothelial cells and microglia, may have been included in previous reports. By quantifying EdU^+^ cells that co-labeled for Pax6 we increased the specificity and sensitivity to changes in proliferation in CE cells, which includes the RSC population. In addition, we observed a significant number of EdU^+^CD68^+^ microglia/macrophages in the CE and showed that different combinations of Noggin, anti-sFRP2, and growth factors had differential off-target effects on the proliferation of endothelial cells, reinforcing the importance of evaluating cell-type-specific markers in the RSC niche.

No change in the number of RSC spheres derived from the CE was observed after FINS treatment, indicative of an asymmetric mode of RSC division [46, 58, 59]. In contrast, individual anti-sFRP2 or Noggin each increased RSC sphere number, signifying symmetric expansion of RSCs in vivo. This is concordant with previous studies that have characterized various factors with different influences on RSC mode of division. For example, in vitro, RSC symmetric expansion can be promoted by exogenous Wnt ligands [60], Notch ligands [46] and PEDF [61], while asymmetric self-renewal of RSCs is dependent on FGF and Notch signaling [46]. In vivo, an expanded RSC population has been demonstrated in mutant mice with reduced populations of NR progenitors (*Chx10*^*orj/orj*^) or RPE progenitors (*Mitf*^*mi/mi*^), suggesting non-cell autonomous signals can regulate the size of the RSC pool [62]. Another study observed only asymmetric cell divisions in the CE of rats in response to intravitreal injections of FGF and Insulin [53], which also is the preferential mode of division of RSCs in the Zebrafish CMZ [63]. Furthermore, both Wnt signaling and BMP signaling are known to crosstalk with one another, as well as with other stem cell regulating pathways, including Notch and Hedgehog [25, 64–66]. Thus, it is likely the different effects on RSC number of the various combinations of exogenous factors used in this study is determined by which signaling pathways predominate under each condition and their particular influences on RSC mode of division. Alternative to RSC mode of division modulating sphere number, it is possible that a subset of RSCs remain quiescent and do not generate spheres in vitro unless first stimulated in vivo. Indeed, other endogenous stem cells, such as neural stem cells in the brain, contain subpopulations in quiescent and activated states which have different colony forming ability in vitro [67–70]. Recent experiments in our lab have generated evidence that endogenous RSCs may also include a subpopulation that are primed to proliferate with a greater propensity for sphere formation in vitro (submitted for publication). However, resolving the active signaling pathways in RSCs or distinct RSC subpopulations in vivo remains challenging given that RSCs are rare cells with no known unique molecular markers.

Since a specific marker for RSCs is not yet known, we investigated the possibility of lineage labeling the entire CE, which includes the RSC population. We observed that *Msx1*-mediated reporter expression can thoroughly label the adult mouse CE. This corresponds with previous studies showing *Msx1* expression throughout the CE during embryonic and early postnatal development [24, 71]. In contrast, Belanger et al. (2016) reported that late embryonic *Msx1* is restricted only to the proximal CE that is continuous with the peripheral neural retina. However, they successfully used the *Msx1-Cre*^*ERT2*^ mouse line for lineage tracing in the embryonic mouse eye and demonstrated retinal neurogenesis from the CE during development [21]. Contrary to our results, the authors stated that their *Msx1* reporter did not label the pigmented CE, and thus, likely did not label CE-RSCs. Here, we report the first evidence *Msx1* is expressed in adult mouse RSCs via the lineage labeling of clonal RSC-derived spheres. Furthermore, FACS revealed that only *Msx1*-labeled pigmented CE cells give rise to clonal RSC spheres. Thus, these results suggest Msx1 is not only expressed in the pigmented CE and RSCs but may even regulate RSC function. Indeed, Msx1 is known to regulate proliferation, differentiation and regenerative processes in other adult stem cells and tissues [72–75]. Also notable, BMPs and Wnts have been proposed to cooperate to pattern the CE by promoting *Msx1* expression [24, 32]. Thus, better resolution of the relationship between BMP, Wnt and Msx1 in RSCs and the adult CE could provide further insight into the regulation of RSC quiescence in the adult mammalian eye. Another difference between our study and the Belanger et al. study is that they did not detect any *Msx1* lineage labeled cells in the adult retina when tamoxifen was injected at comparable adult mouse timepoints (P28 or P37). Albeit, only our Naïve control group is comparable to the mice in the Belanger study and it was extremely rare to find *Msx1*-labeled cells in the retinas of Naïve mice. Plus, when cells were detected, they were immediately adjacent to the CE in the peripheral tip of the retina (Additional file 1: Fig S12). Also, our study appeared to produce more thorough labeling of the CE. Our tamoxifen induction paradigm used the same 180 mg/kg dose but included one additional day of injection (4 vs 3 total injections), which may have resulted in this difference. Further, Belanger et al. analyzed the retina 1 week post-tamoxifen, while we analyzed after 4 weeks. Thus, these differences in experimental parameters may have led to our different findings. Another important contrast is that we observed *Msx1* lineage labeling in eye tissues other than the CE (Additional file 1: Figure S16). Because the labeling in these tissues is either not closely apposed to the retina or not pronounced, we do not believe they would confound CE lineage tracing. Yet, these other tissues cannot be completely ruled out as potential sources of *Msx1*-tdTomato^+^ cells in the retina. Thus, more in-depth profiling of *Msx1* lineage labeled cells in the retina is required to be certain of their tissue of origin and verify that *Msx1*-tdTomato^+^ cells that co-label with retinal neuron markers are truly retinal neurons.

Some studies have reported proliferation, and even expression of retinal neuron markers, in the adult mammalian CE following injury [76–80]. We found only minor evidence of CE proliferation in response to MNU injury and did not find any evidence of retinal neuron markers within the CE. However, MNU injury did induce some CE cell migration into the retina where the majority of *Msx1*^+^ cells co-labeled for photoreceptor (recoverin) or RGC (Brn3a) markers. Furthermore, FINS with MNU injury resulted in similar migration and fewer Brn3a^+^*Msx1*-tdTomato^+^ co-labeled cells than FINS injection alone. In the xenopus and zebrafish CMZ, the influence of injury on retinal stem and progenitor cell proliferation, migration and differentiation arises from damage-liberated trophic factors and cytokines [47, 81, 82]. Thus, damage-liberated factors could modulate proliferation and differentiation in the reactivated adult mammalian CE as well. Another consideration is that injury-induced reactive gliosis in the retina, which is caused by MNU-mediated retinal degeneration [83], has been shown to decrease migration, maturation and survival of transplanted photoreceptors [84]. Thus, a less severe injury model than MNU may also change migration and differentiation outcomes. However, it is important to emphazise that this study only surveyed single markers of retinal cell types via IHC and did not attempt to thoroughly and definitively establish differentiation of *Msx1*-tdTomato^+^ CE cells into retinal neurons. To properly establish differentiation will be the goal of future studies and will require much more in-depth characterization of *Msx1*-tdTomato^+^ cells using multiple cell-specific markers, genetic reporters, single cell RNAseq and functional assays, like electrophysiology. In addition, prior studies in the post-hatch chick ciliary marginal zone have shown that different exogenous growth factors result in different types of cells produced by retinal progenitors at the CMZ [85], while our lab has demonstrated that exogenous factors can be used to influence the cell fate specification of retinal stem and progenitor cells in vitro [5, 6]. Therefore, it is possible that different combinations of FINS components and/or injury could bias differentiation to particular cell types in the adult mammalian CE but this is yet to be determined.

It also remains to be investigated whether the activation of CE-RSC proliferation, migration and putative differentiation observed in this study might have a functional impact toward restoring vision. If new retinal neurons are being generated, relatively few cells per eye may be needed to improve visual function, as a previous study found the survival of ~ 350 RSC-derived rod photoreceptors in the retina after transplantation was sufficient to increase the pupillary light response in blind mice [20], while a survival rate of as few as 10–200 cells in the retina has been shown to improve visual function after retinal progenitor transplantation [86]. Nonetheless, it is likely the strategy employed in this study can be improved to generate an even greater stimulation of endogenous RSCs and increase the likelihood of restoring visual function. This study employed an acute, three-day intravitreal injection paradigm and there was evidence the effects of Noggin and anti-sFRP2 largely subsided 24 h after injection (Fig. 1H-K). Furthermore, we used a variety of factors that likely have different pharmacokinetics in the vitreous [87]. Therefore, follow up studies using biomaterials, such as polymeric microparticles or hydrogels, to achieve controlled and sustained release of FINS components into the vitreous may elicit more robust and consistent proliferation, migration and neurogenesis in the RSC niche [88, 89]. Yet, although we did not see any evidence of dysplasia or tumor-like masses in our analyses, this is a valid concern that should continue to be assessed with further adaptations of the stimulation protocol, such as prolonged delivery or a changes in formulation.

## Conclusions

In summary, this study demonstrates that antagonism of putative RSC quiescence factors, BMP or sFRP2, can stimulate the proliferation and symmetric expansion of endogenous RSCs in the adult mammalian eye. Furthermore, BMP and sFRP2 antagonism, growth factor stimulation, chemically-mediated photoreceptor degeneration, or combinations thereof, induces CE cells in the RSC niche to proliferate and migrate into the retina, where a significant subpopulation express markers of mature photoreceptors and retinal ganglion cells. Together, these results evince that adult mammalian RSCs may be a latent source of endogenous retinal neurons that could be stimulated to regenerate the retina to treat otherwise irreversible retinal degenerative diseases. However, future studies will be required to determine if adult RSC-derived progeny can form functional retinal neurons that properly integrate with existing retinal circuitry and have the capacity to resotre visual function. If such functional properties can be demonstrated, then stimulation of adult RSCs could become a viable means to induce endogenous retinal regeneration and reverse vision loss caused by retinal damage or degenerative diseases.

## Supplementary Information

**Additional file 1.** **Supplementary Figures.** Figure S1–Figure S16. **Supplementary Table.** Table S1.

## Data Availability

The data that support the findings of this study are available from the corresponding author upon reasonable request.

## Abbreviations

BMP: Bone morphogenetic protein
CB: Ciliary body
CE: Ciliary epithelium
CMZ: Ciliary marginal zone
DMSO: Dimethylsulfoxide
EdU: 5-Ethynyl-2’-deoxyuridine
EtOH: Ethanol
FACS: Fluorescence-activated cell sorting
FBS: Fetal bovine serum
FGF: Fibroblast growth factor
FH: FGF + Heparin
FINS: FGF2, Insulin, Noggin, anti-sFRP2
ICC: Immunocytochemistry
IGF: Insulin-like growth factor
IHC: Immunohistochemistry
MNU: *N*-Methyl-*N*-nitrosourea
MSX1: Muscle segment homeobox 1
NC: Naïve control
NPE: Non-pigmented epithelium
NR: Neural retina
ONL: Outer nuclear layer
OPE: Outer pigmented epithelium
PBS: Phosphate buffered saline
RGC: Retinal ganglion cell
RPE: Retinal pigmented epithelium
RSC: Retinal stem cell
SFM: Serum-free media
sFRP2: Secreted frizzled-related protein 2
