# Supplementary material for: Activation of adult mammalian retinal stem cells in vivo via antagonism of BMP and sFRP2
Source: Stem Cell Res Ther. 2021 Oct 30;12:560. doi: 10.1186/s13287-021-02630-0 (PMC8557620; doi:10.1186/s13287-021-02630-0)
Supplement: Supplementary file 1 — Additional file 1. Supplementary Figures. Figure S1–Figure S16. Supplementary Table. Table S1. [file 13287_2021_2630_MOESM1_ESM.docx]

**SUPPLEMENTARY INFORMATION**

**Activation of adult mammalian retinal stem cells in vivo via antagonism of BMP and sFRP2**

Kenneth N. Grisé, Brenda L.K. Coles, Nelson X. Bautista, Derek van der Kooy

**SUPPLEMENTARY FIGURES**

Figure S1 – Figure S16.

**SUPPLEMENTARY TABLES**

Table S1.


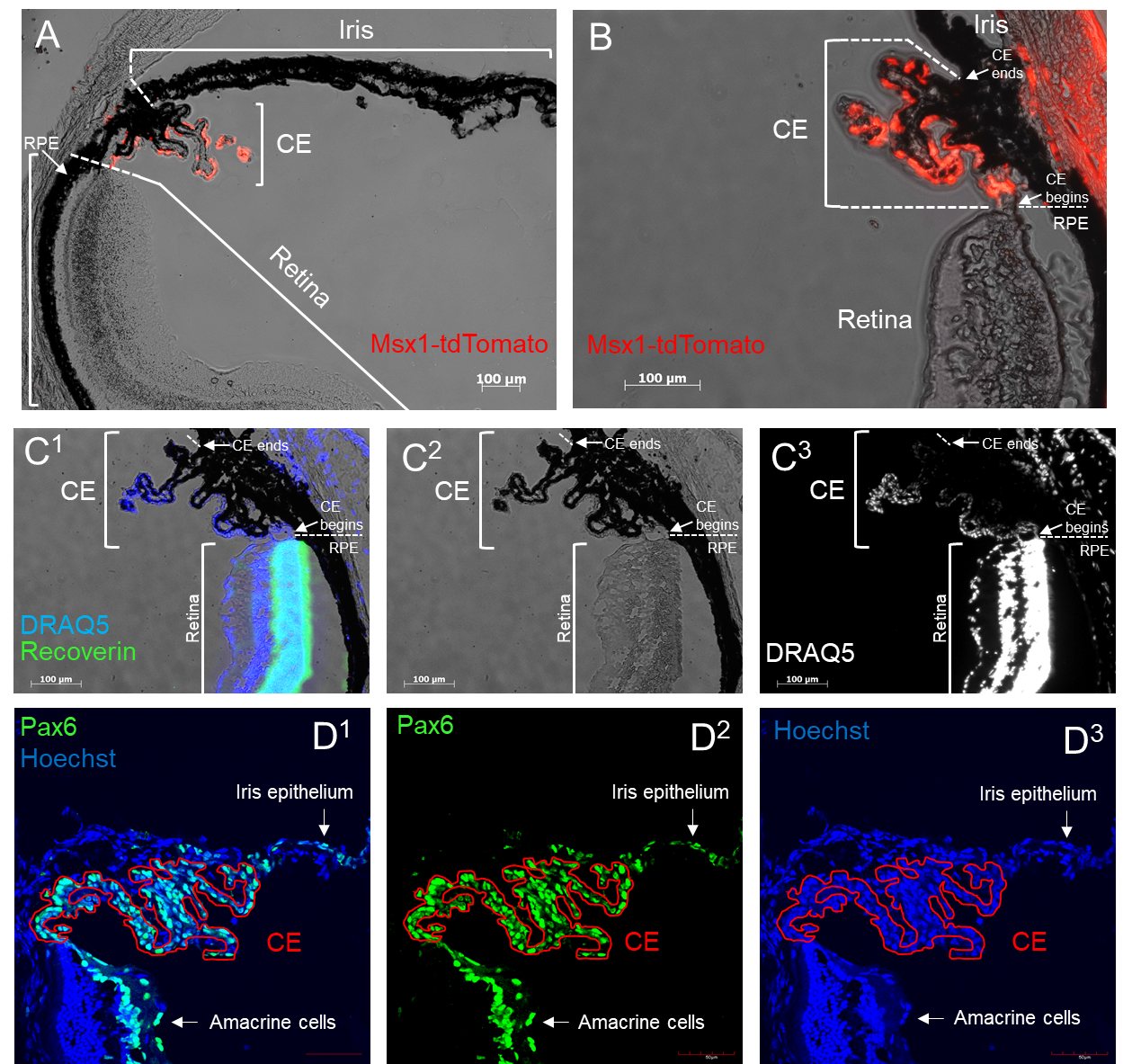


**Supplementary Figure 1.** The morphological features and cell markers that delineate the ciliary epithelium (CE) from the neural retina (NR), retinal pigmented epithelium (RPE), iris, and surrounding tissue.

**(A-B)** Brightfield and fluorescence overlay images of Msx1-Cre driven tdTomato expression in naïve adult mouse eye sections. *Msx1* is expressed specifically in the CE under normal conditions. This can distinguish the CE from the NR, RPE, iris and other cells of the ciliary body tissue.

**(C)** Brightfield and fluorescence overlay images of nuclear marker DRAQ5 and photoreceptor-specific marker, Recoverin – which labels the entire outer nuclear layer (ONL) of the retina and is useful to demarcate where the NR ends, and the CE begins. In C^3^, where “CE begins” is labeled, it is observable how the laminated NR and the multiple-nuclei-thick ONL is contiguous with the single cell layer of the inner CE.

**(D)** Pax6 labels CE cells and enables the distinction of CE cells from other cells in the ciliary body. As indicated, Pax6 also labels amacrine cells in the retina and the epithelium of the iris. The red outline is an example of how the CE was traced to separate it from surrounding tissues and calculate the CE area for quantification.

Dashed lines indicate border regions between the CE and NR, CE and RPE, or CE and iris. 10µm-thick sections.

**
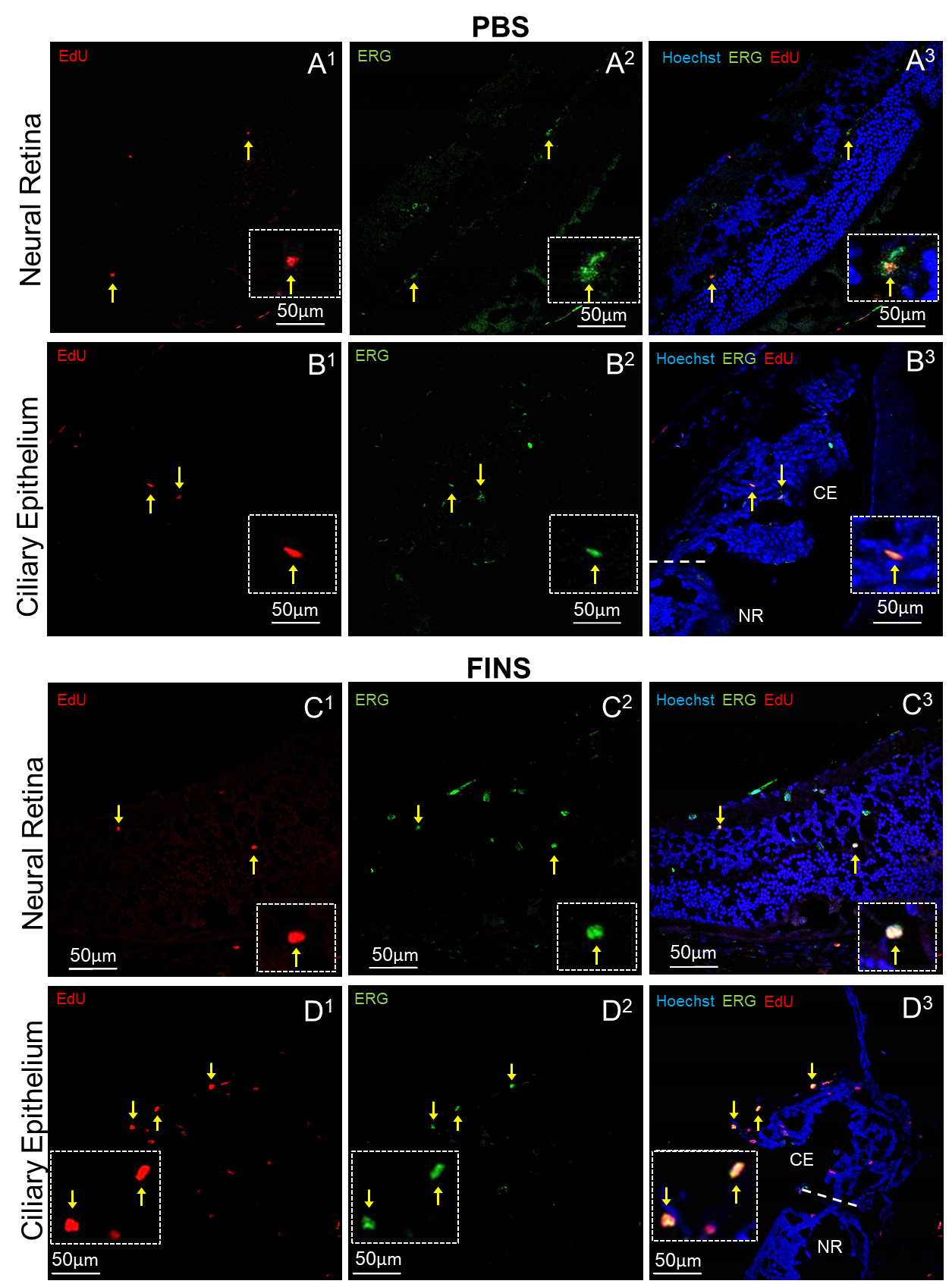
**

**Supplementary Figure 2. Some EdU^+^ cells in the ciliary epithelium and neural retina co-labeled for endothelial cell marker ERG.**

**(A-B)** EdU and ERG labeling in the **(A)** neural retina and **(B)** ciliary epithelium of PBS treated eyes.

**(C-D)** EdU and ERG labeling in the **(C)** neural retina and **(D)** ciliary epithelium of FINS treated eyes.

Endothelial nuclei vary in shape from rounded **(B)** to more elongated **(C)** and sometimes multiple are observed in close association **(A)**. Hoechst was used to label all nuclei. Dashed line box indicates high magnification inset. Straight dashed line indicates ciliary epithelium (CE) neural retina (NR) border. 10µm thick sections.

FINS = FGF2 + Insulin + Noggin + anti-sFRP2 combined intravitreal injection; PBS = PBS intravitreal injection.

**
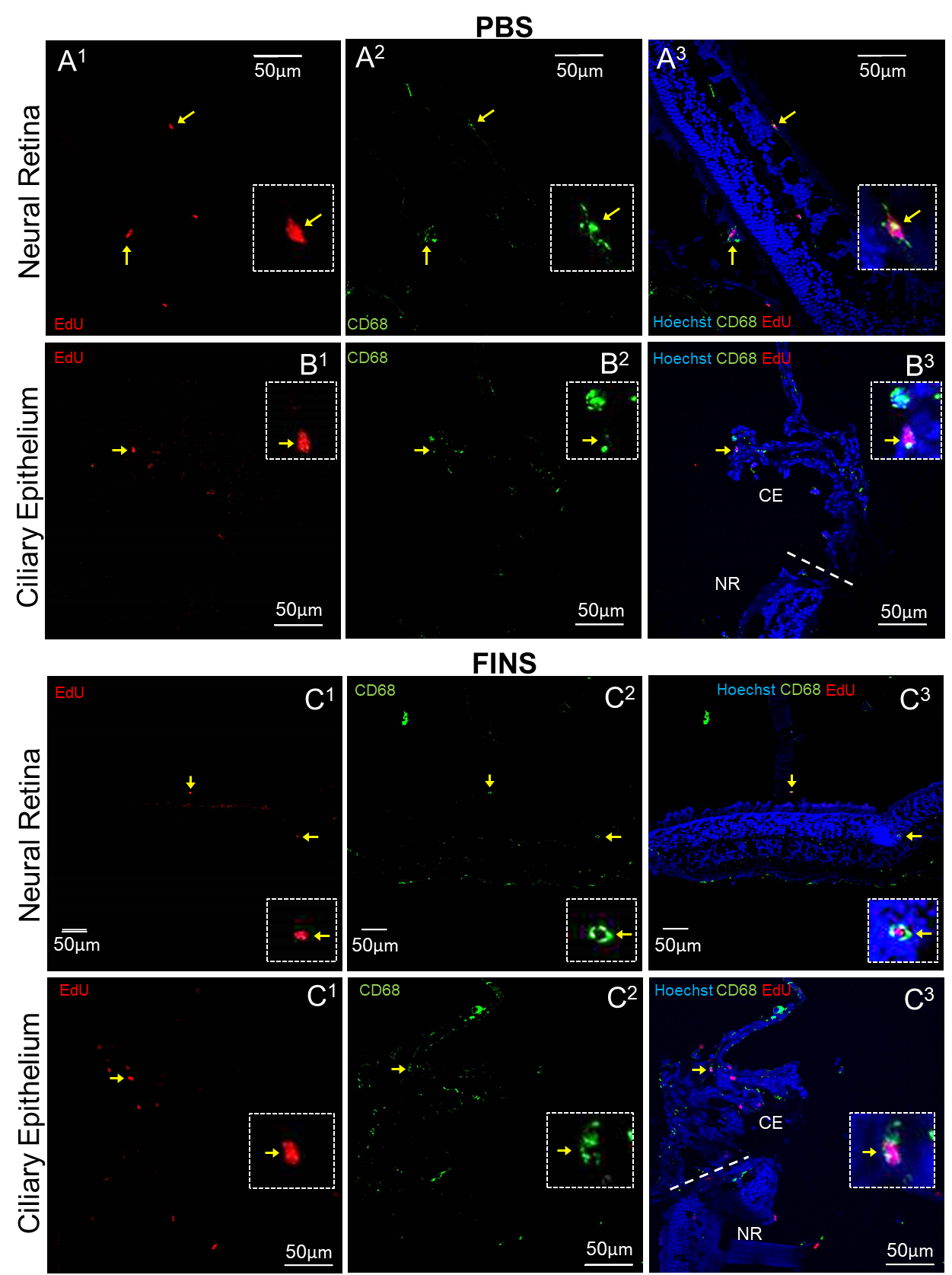
**

**Supplementary Figure 3. Some EdU^+^ cells in the ciliary epithelium and neural retina co-labeled for microglia/macrophage cell marker CD68.**

**(A-B)** EdU and CD68 labeling in the **(A)** neural retina and **(B)** ciliary epithelium of PBS treated eyes.

**(C-D)** EdU and CD68 labeling in the **(C)** neural retina and **(D)** ciliary epithelium of FINS treated eyes.

Hoechst was used to label all nuclei. Dashed line box indicates high magnification inset. Straight dashed line indicates ciliary epithelium (CE) neural retina (NR) border. 10µm thick sections.

FINS = FGF2 + Insulin + Noggin + anti-sFRP2 combined intravitreal injection; PBS = PBS intravitreal injection.

‘


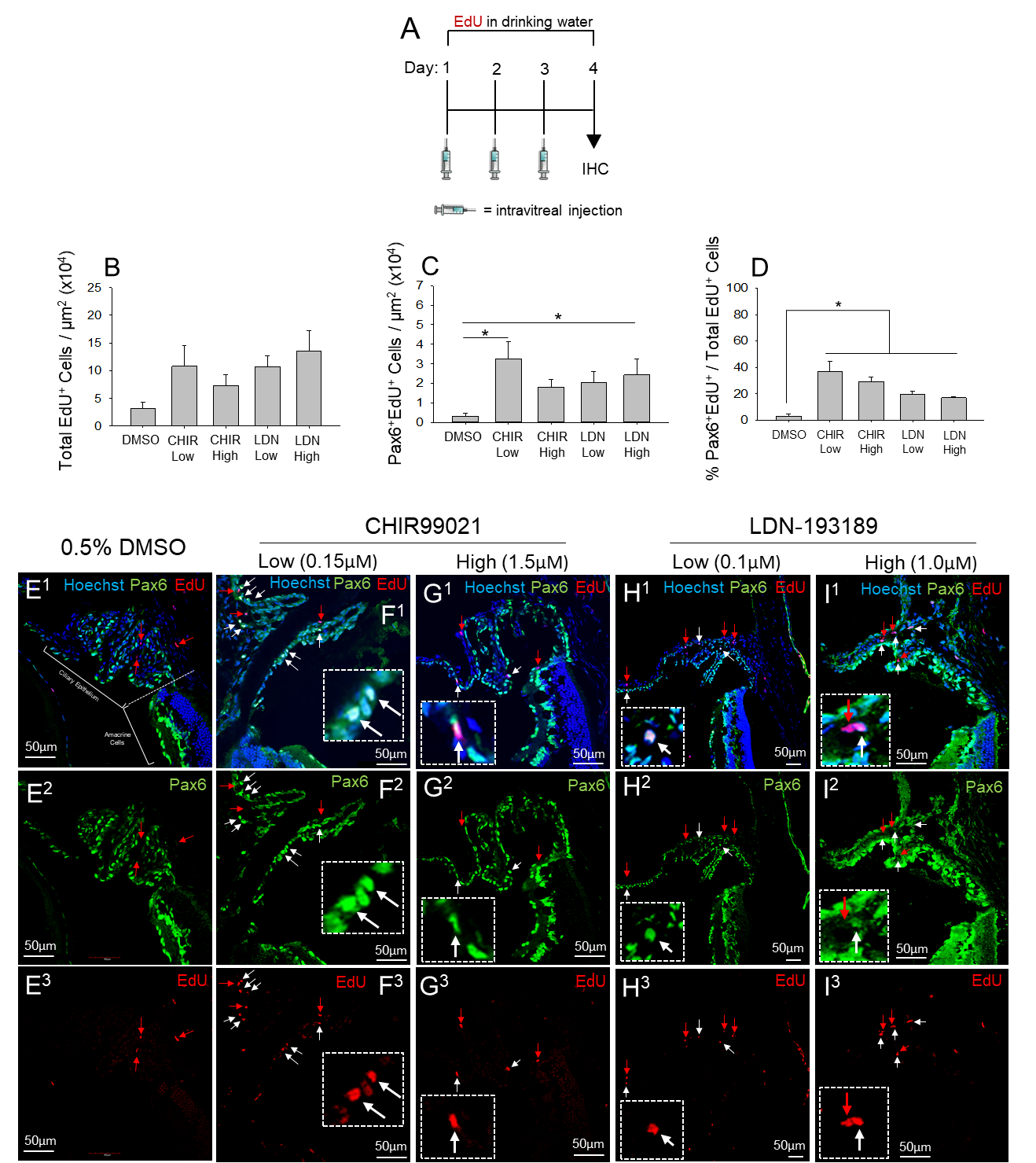


**Supplementary Figure 4.** **Modulation of downstream BMP and Wnt signaling mediates CE proliferation.**

**(A**) Schematic of the intravitreal injection paradigm followed by endpoint IHC. Mice received one intravitreal injection per day for three days while EdU was delivered via the drinking water continuously until the Day 4 endpoint. Injections consisted of 0.5% DMSO control, CHIR99021 or LDN-193189.

**(B)** Quantification of EdU cell number in the CB normalized by area in eyes treated with the indicated conditions. N=5-6 eyes per group. Data are Mean ± SEM.

**(C)** Quantification of Pax6^+^EdU^+^ co-labeled cells relative to total CE area in eyes treated with PBS vehicle or indicated factors (one-way ANOVA F(_4,23_)=3.23, p=0.031; N=5-6 eyes per group). Holm-Sidak posthoc test, * = p<0.05. Data are Mean ± SEM.

**(D)** Percent of total EdU-positive cells in the CB that co-labeled for CE marker Pax6. (one-way ANOVA F(_4,22_)=10.58, p<0.001; N=5-6 eyes per group). Holm-Sidak posthoc test, * = p<0.05. Data are Mean ± SEM.

**(E-I)** Pax6 immunostaining and EdU labeling in the ciliary body. Hoechst stain was used to label all nuclei. White arrows indicate Pax6^+^EdU^+^ double-positive cells. Red arrows indicate EdU^+^ only cells. Dashed line box indicates high magnification inset. Straight dashed line indicates ciliary epithelium (CE) neural retina (NR) border. 10µm-thick sections.

**
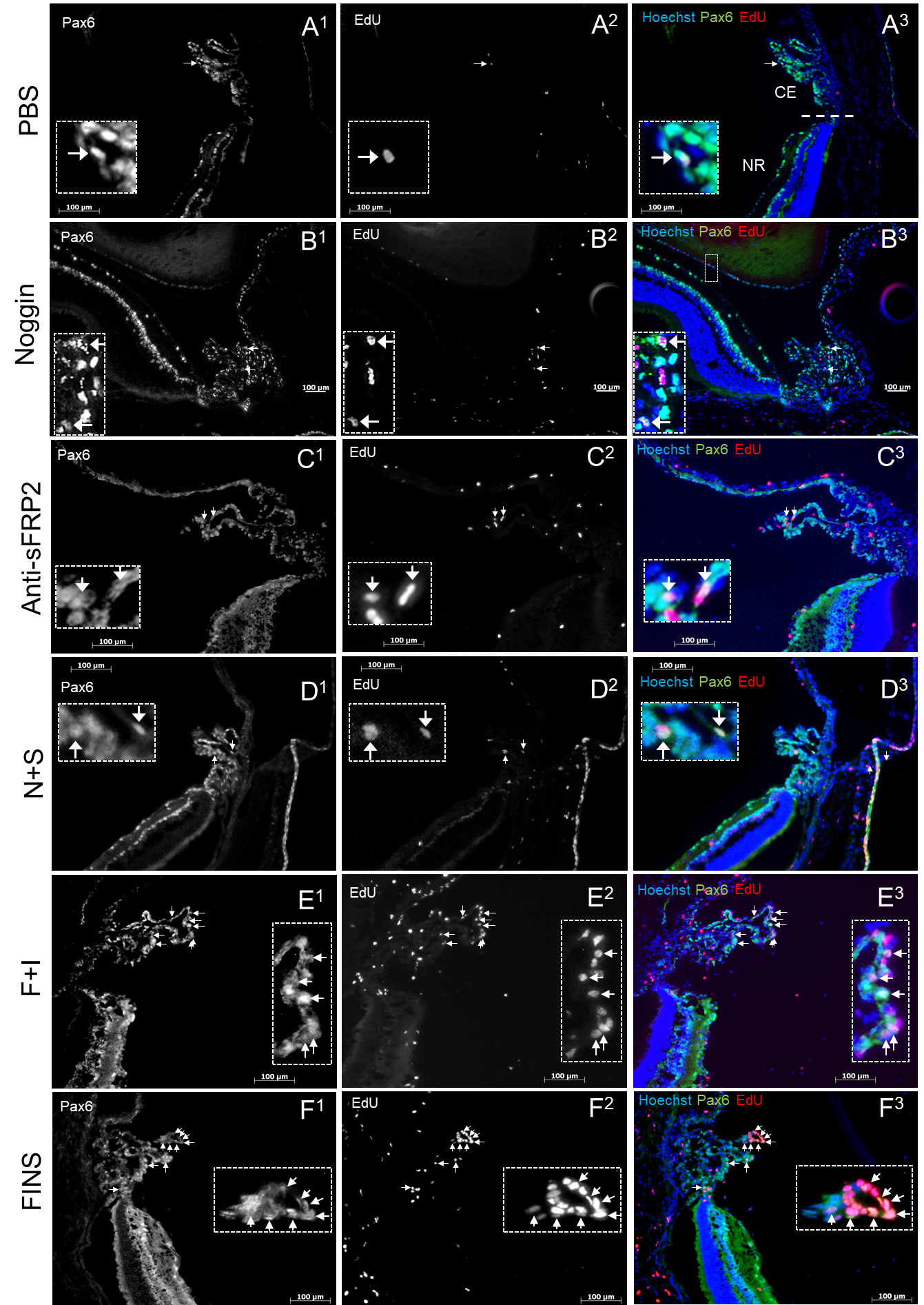
**

**Supplementary Figure 5.** Day 4 multichannel images of the representative IHC images in Figure 2.

**(A-F)**. Pax6 immunostaining and EdU labeling in the ciliary epithelium and peripheral retina of eyes injected with PBS vehicle or indicated factors at Day 4. Hoechst stain was used to label all nuclei. White arrows indicate Pax6^+^EdU^+^ double-positive cells. Dashed line box indicates high magnification inset. Straight dashed line indicates ciliary epithelium (CE) neural retina (NR) border. 10µm-thick sections.

N+S=Noggin+anti-sFRP2 combined, F+I=FGF2+Insulin combined, FINS=FGF2+Insulin+Noggin+anti-sFRP2 combined.

**
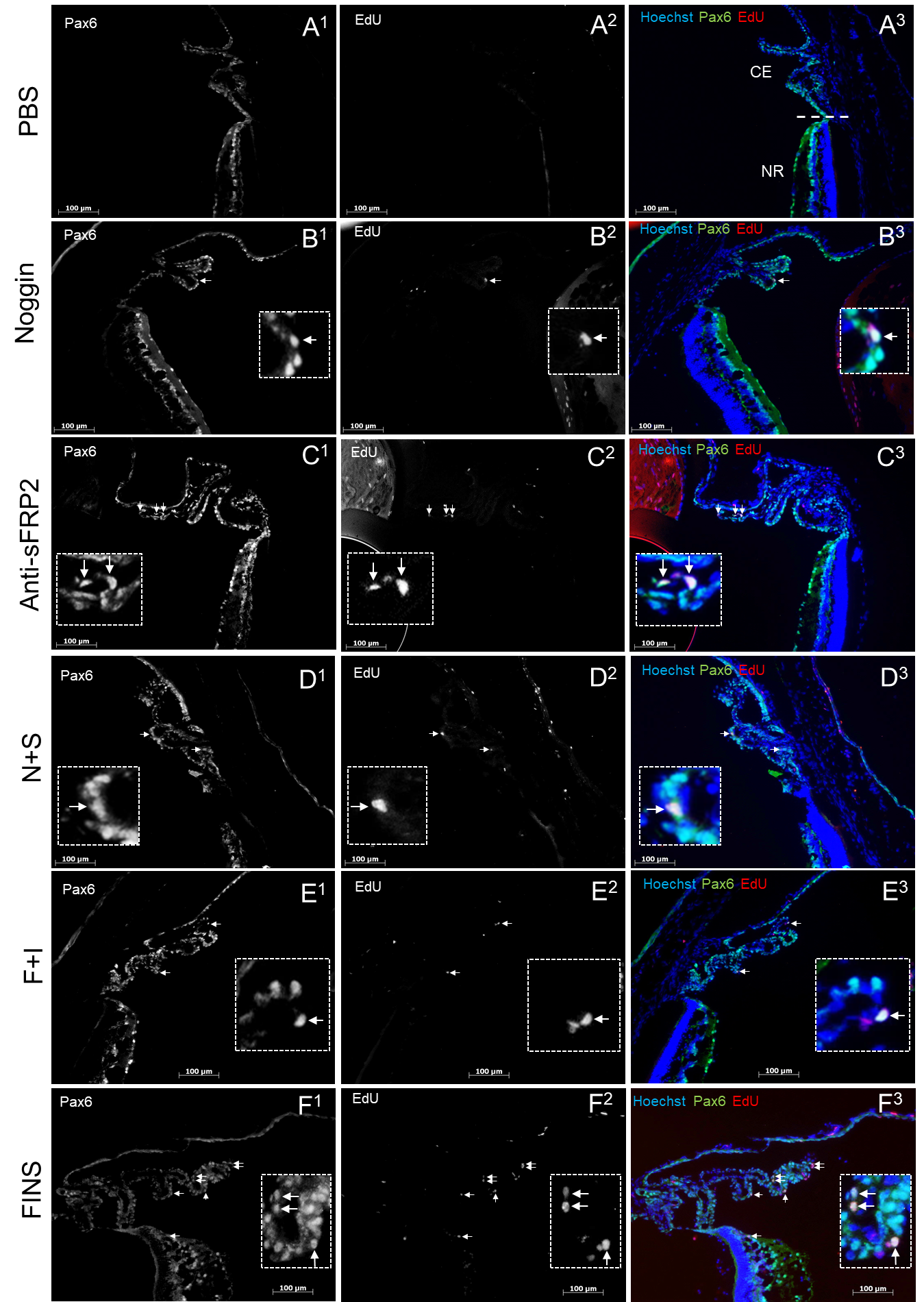
**

**Supplementary Figure 6.** Day 31 multichannel images of the representative IHC images in Figure 2.

**(A-F)**. Pax6 immunostaining and EdU labeling in the ciliary epithelium and peripheral retina of eyes injected with PBS vehicle or indicated factors at Day 31. Hoechst stain was used to label all nuclei. White arrows indicate Pax6^+^EdU^+^ double-positive cells. Dashed line box indicates high magnification inset. Straight dashed line indicates ciliary epithelium (CE) neural retina (NR) border. 10µm-thick sections.

N+S=Noggin+anti-sFRP2 combined, F+I=FGF2+Insulin combined, FINS=FGF2+Insulin+Noggin+anti-sFRP2 combined.

**
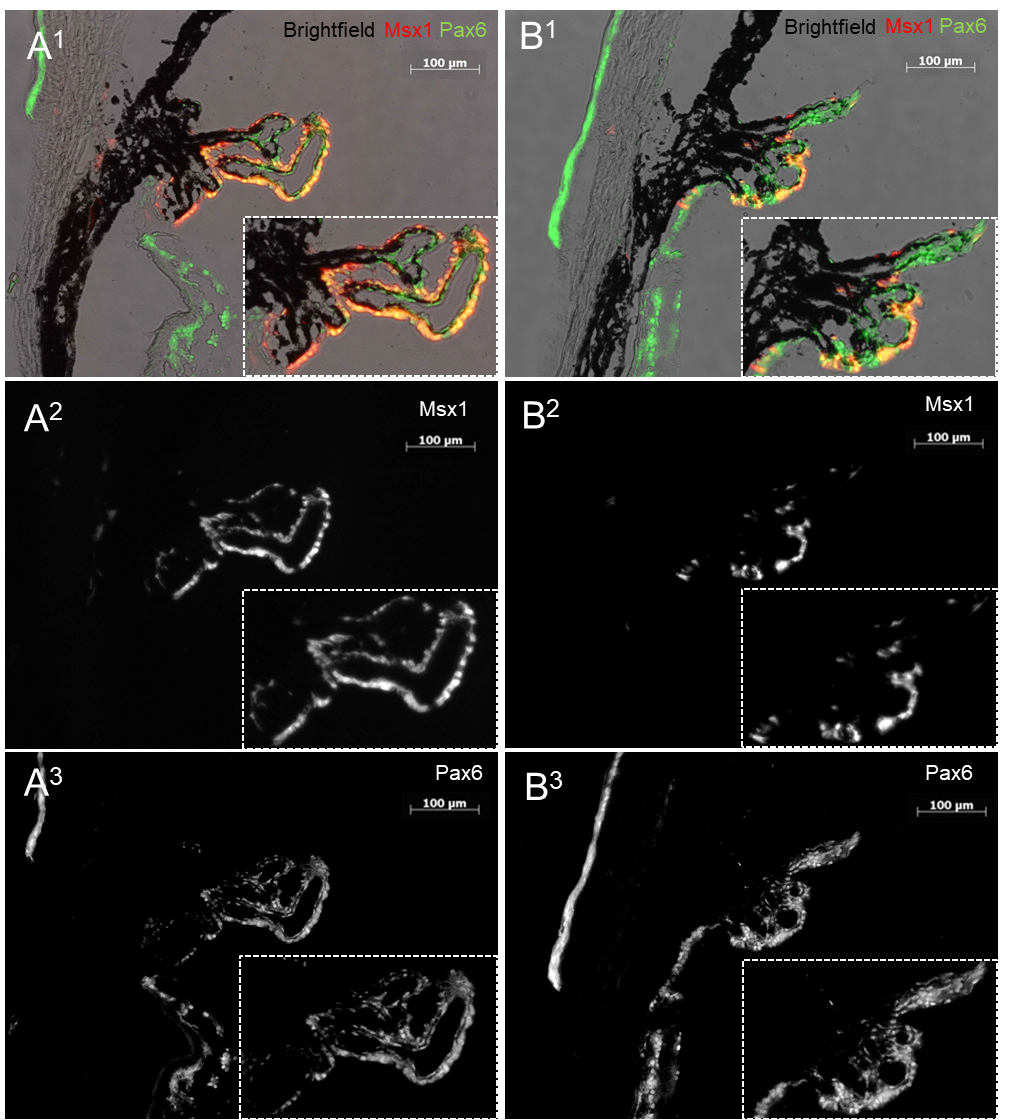
**

**Supplementary Figure 7. Pax6 and Msx1 labeling overlaps**

**(A)** Thorough penetrance of Msx1 labeling in the CE completely overlaps with Pax6 labeling.

**(B)** Incomplete penetrance of Msx1 labeling in the CE still overlaps with Pax6 where present.

Dashed line box indicates high magnification inset. 10µm thick sections.


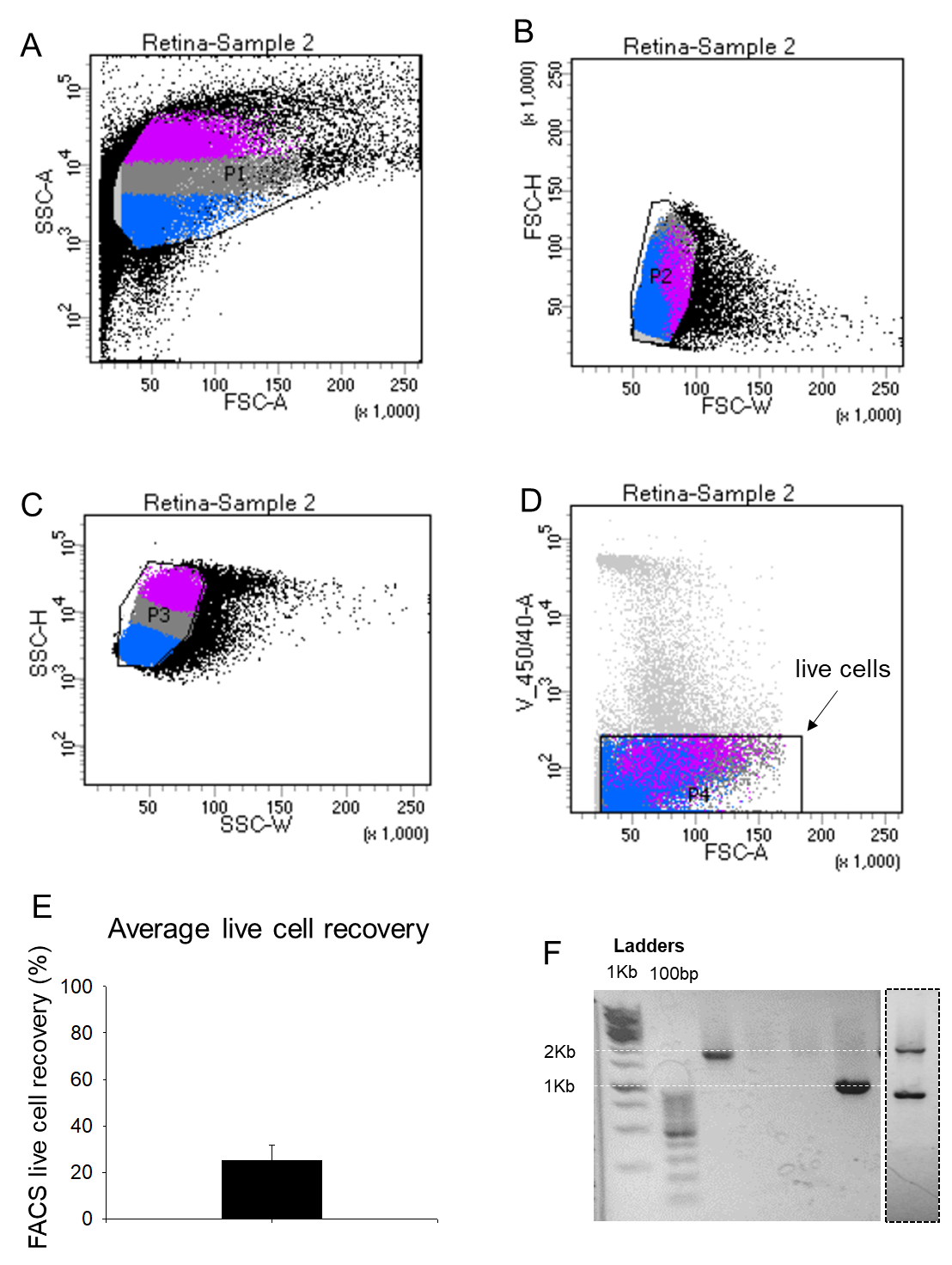


**Supplementary Figure 8. Initial FACS gating to select live cells and the stop codon genotype of post-FACS RSC spheres.**

**(A-D)** Representative FACS gating plots for CE cells derived from Msx1-Cre^ERT2^;Rosa26-tdTomato mice (A-C) Forward and side scatter gating. (D) DAPI based live-dead cell gating.

**(E)** Average live cell recovery from as a percentage of total events detected. N=3. Data is mean ± SEM.

**(F)** Cropped, PCR gel for DNA extracted from single RSC spheres, which was amplified using primers spanning the floxed stop codon region of the Rosa26-tdTomato reporter construct. A 2Kb band indicates the presence of the stop codon. A 1Kb band indicates excision of the stop codon. For 20/21 of spheres tested, a single 1Kb or 2Kb DNA band was detected, indicating the presence of a single genotype for all cells in the sphere. The one instance where two bands were detected in one lane has been cropped from the gel and inserted for reference, indicated by the dashed outline.


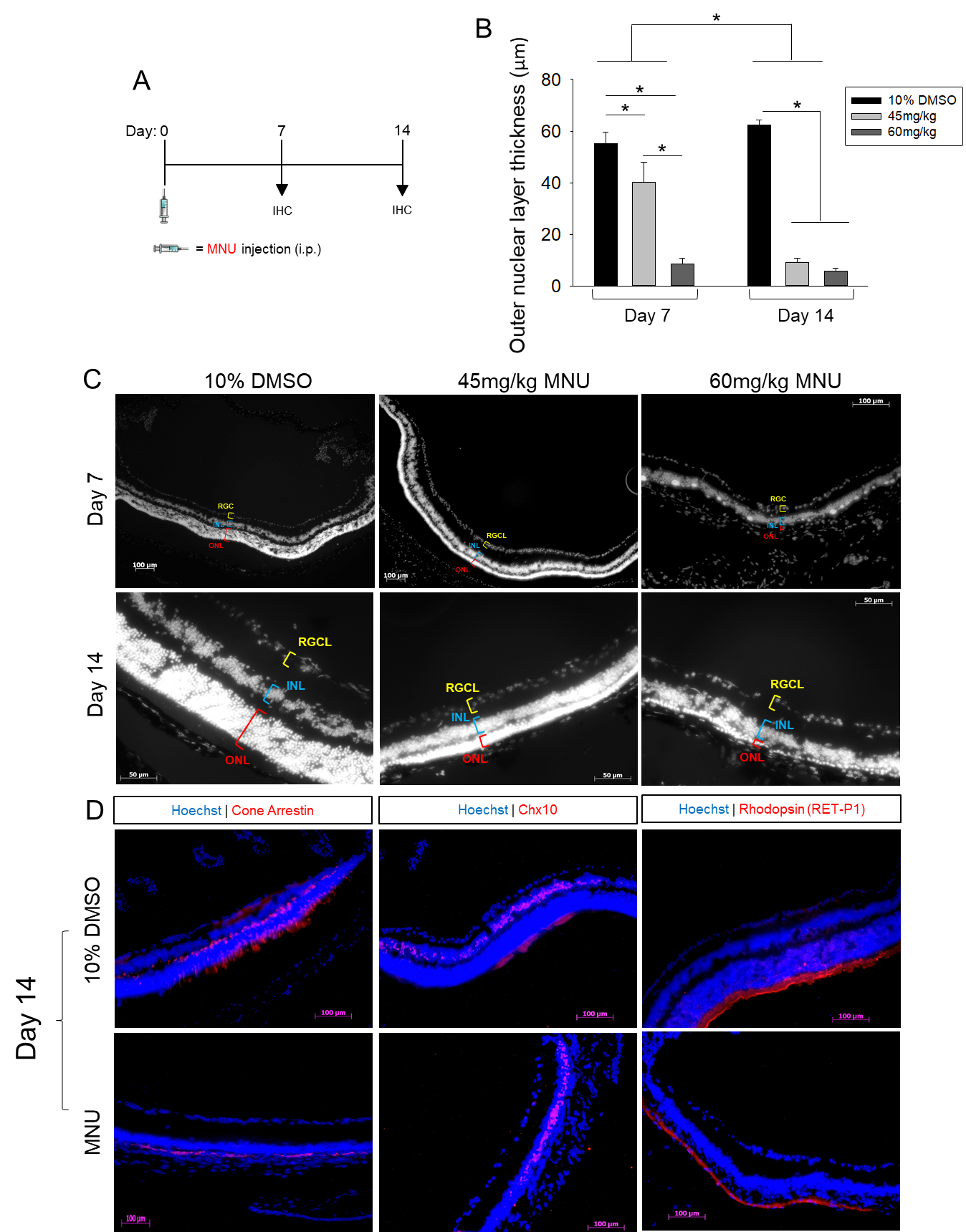


**Supplementary Figure 9. *N*-methyl-*N*-nitrosourea induces tunable photoreceptor degeneration**

**(A**) Schematic of intraperitoneal injection of 45mg/kg or 60mg/kg MNU followed by IHC of eyes 7 days and 14 days after injection.

**(B)** Outer nuclear layer thickness from mice treated with MNU or 10% DMSO. (two-way ANOVA F(_2,29_)=12.15, p<0.001; N=5-6 eyes per group). Holm-Sidak posthoc test, * = p<0.05. Data are mean ± SEM.

**(C)** Hoechst nuclear staining of retinas from mice treated with the indicated conditions at Day 7 and Day 14. RGC = retinal ganglion cell layer, INL = inner nuclear layer, ONL = outer nuclear layer 10µm-thick sections.

**(D)** Immunostaining for cones (cone arrestin), bipolar cells (Chx10) and rod outer segments (RET-P1) in retinas from mice treated with the indicated conditions at Day 14. Hoechst was used to label all nuclei. 10µm-thick sections.


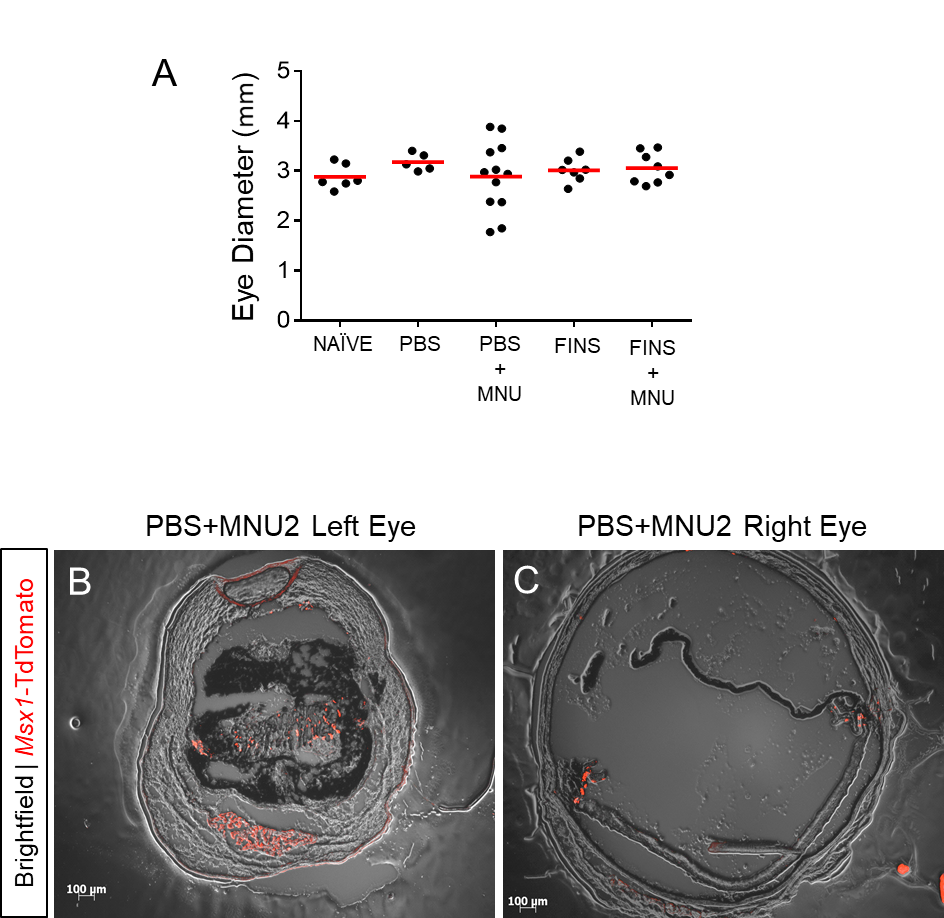


**Supplementary Figure 10. PBS injected eyes with MNU injury occasionally developed gross phthisis bulbi pathology whereas FINS injected eyes with MNU injury did not.**

**(A)** Quantification of mid-sagittal eye diameter. N=5-12 eyes per group. Each data point represents a single eye and mean ± SEM is indicated.

**(B-C)** Brightfield and fluorescence overlay images of Msx1-Cre driven tdTomato expression in PBS+MNU treated eyes from the same mouse with **(B)** whole-eye phthisis bulbi with internal disorganization and **(C)** normal morphology.

10µm-thick sections. Groups included: Naïve control = no injection; PBS = intravitreal PBS; PBS+MNU = intravitreal PBS and i.p. MNU; FINS = intravitreal FINS; FINS+MNU = intravitreal FINS and i.p. MNU. FINS = FGF2 + Insulin + Noggin + anti-sFRP2 combined intravitreal injection.


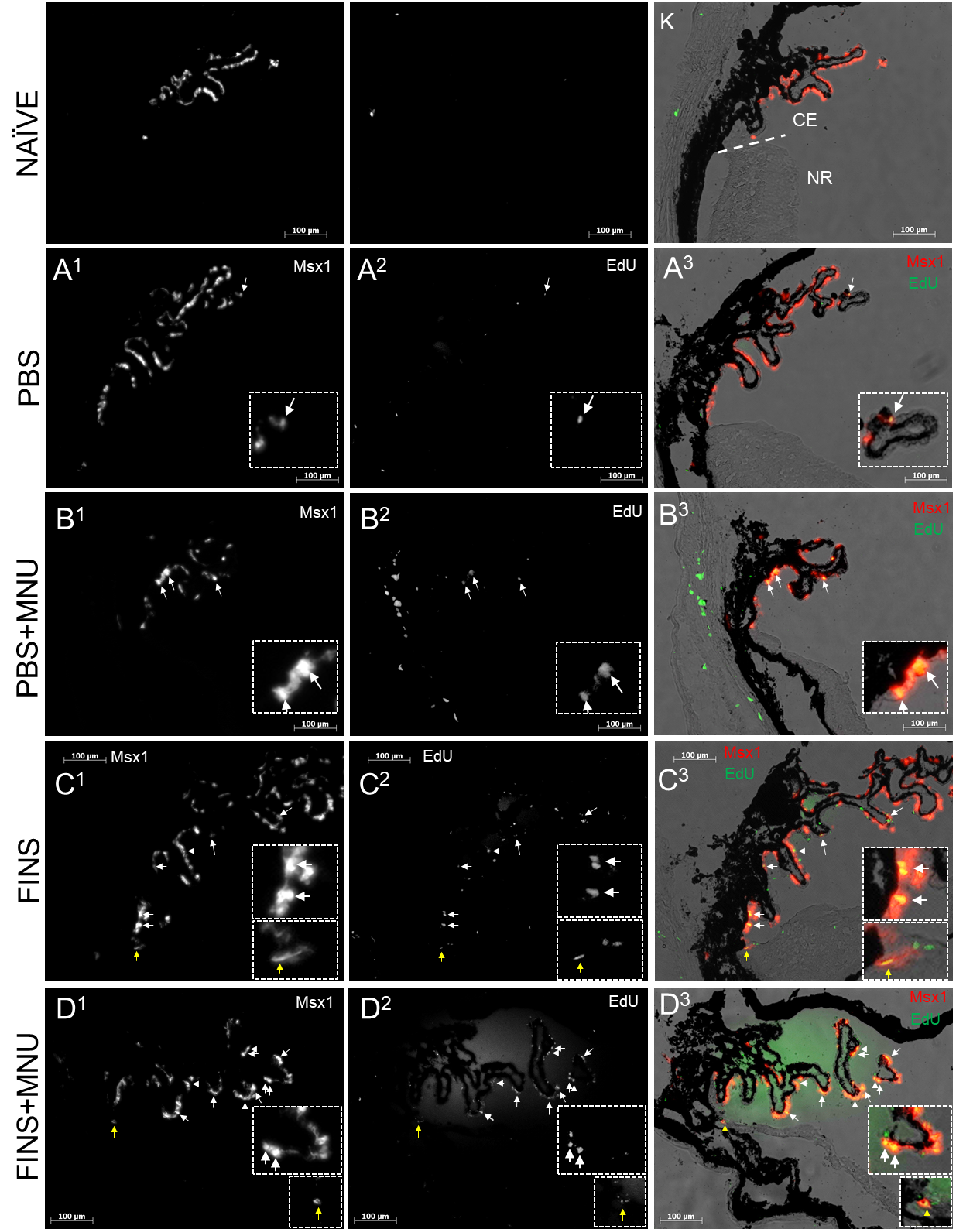


**Supplementary Figure 11.** Multichannel images of the representative Msx1 and Pax6 IHC images in Figure 6.

**(A-D)** Brightfield and fluorescence overlay images of Pax6 immunostaining and Msx1-Cre driven tdTomato expression in eye sections from the indicated conditions. White arrows indicate *Msx1*-tdTomato^+^Pax6^+^ co-labeled cells. Yellow arrowheads indicate *Msx1*-tdTomato^+^EdU^+^ co-labeled cells in the retina. Dashed line box indicates high magnification inset. Dashed line box indicates high magnification inset. Straight dashed line indicates ciliary epithelium (CE) neural retina (NR) border. 10µm-thick sections.

Groups included: Naïve control = no injection; PBS = intravitreal PBS; PBS+MNU = intravitreal PBS and i.p. MNU; FINS = intravitreal FINS; FINS+MNU = intravitreal FINS and i.p. MNU. FINS = FGF2 + Insulin + Noggin + anti-sFRP2 combined intravitreal injection.


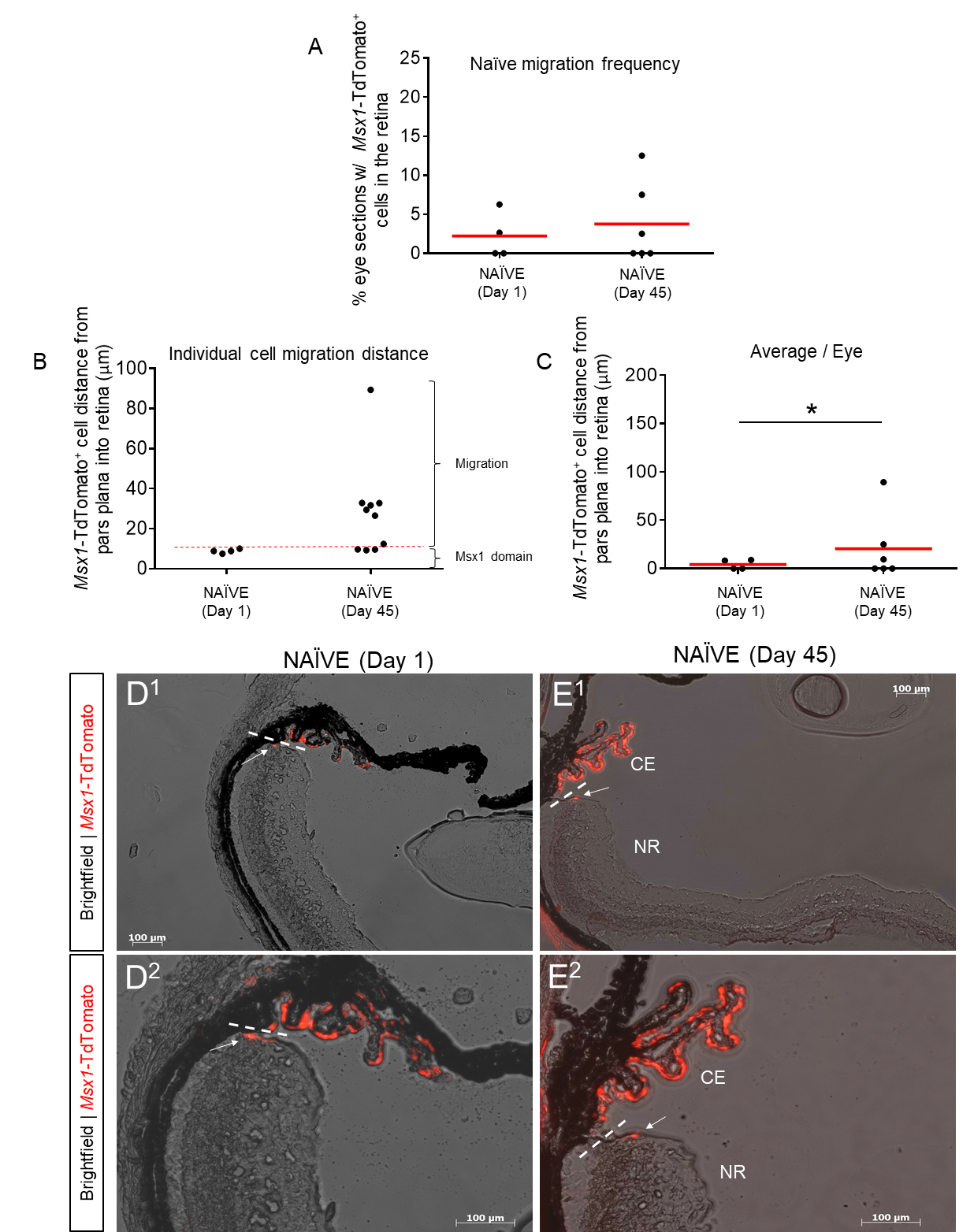


**Supplementary Figure 12. The *Msx1*-tdTomato expression domain of naïve eyes at 1 day and 45 days after tamoxifen induction.**

**(A)** Percent of eye sections with *Msx1*-tdTomato^+^ cells present in retina for naïve Msx1-Cre^ERT2^;Rosa26-tdTomato mice immediately following tamoxifen induction (Day 1) or 45 days after tamoxifen induction (Day 45). The two timepoints were not significantly different (t-test *t*(_8_) = 0.53, p=0.61; Naïve Day 1, N=4, Naïve Day 45, N=6. Each data point represents a single eye and mean ± SEM is indicated.

**(****B-C)** Quantification of the migration distance of *Msx1*-tdTomato^+^ cells into the retina for for naïve Msx1-Cre^ERT2^;Rosa26-tdTomato mice immediately following tamoxifen induction (Day 1) or 45 days after tamoxifen induction (Day 45). **(B)** The migration distance recorded for individual cells. Each data point represents a single cell and mean ± SEM is indicated. Naïve Day 1, N=4 cells from 4 eyes; Naïve Day 45, N=10 cells from 6 eyes. **(C)** Average migration distance per eye. At Day 45, cells were a significantly greater distance into the retina than at Day 1 (t-test *t*(_8_) = 0.91, p=0.0086. Naïve Day 1, N=4, Naïve Day 45, N=6. Each data point represents a single eye. * = p<0.05.

**(D-E)** Brightfield and fluorescence overlay images of Day1 **(D^1^-D^2^)** and Day 45 **(E^1^-E^2^)** Naïve Msx1-Cre^ERT2^;Rosa26-tdTomato mouse eyes with tdTomato^+^ cells in the retina. B^1^ and B^2^ are the same retinal margin at different magnifications. .C^1^ and C^2^ are the same retinal margin at different magnifications. Straight dashed line indicates ciliary epithelium (CE) neural retina (NR) border. White arrows indicate *Msx1*-tdTomato^+^ cells in the retina. 10µm-thick sections.


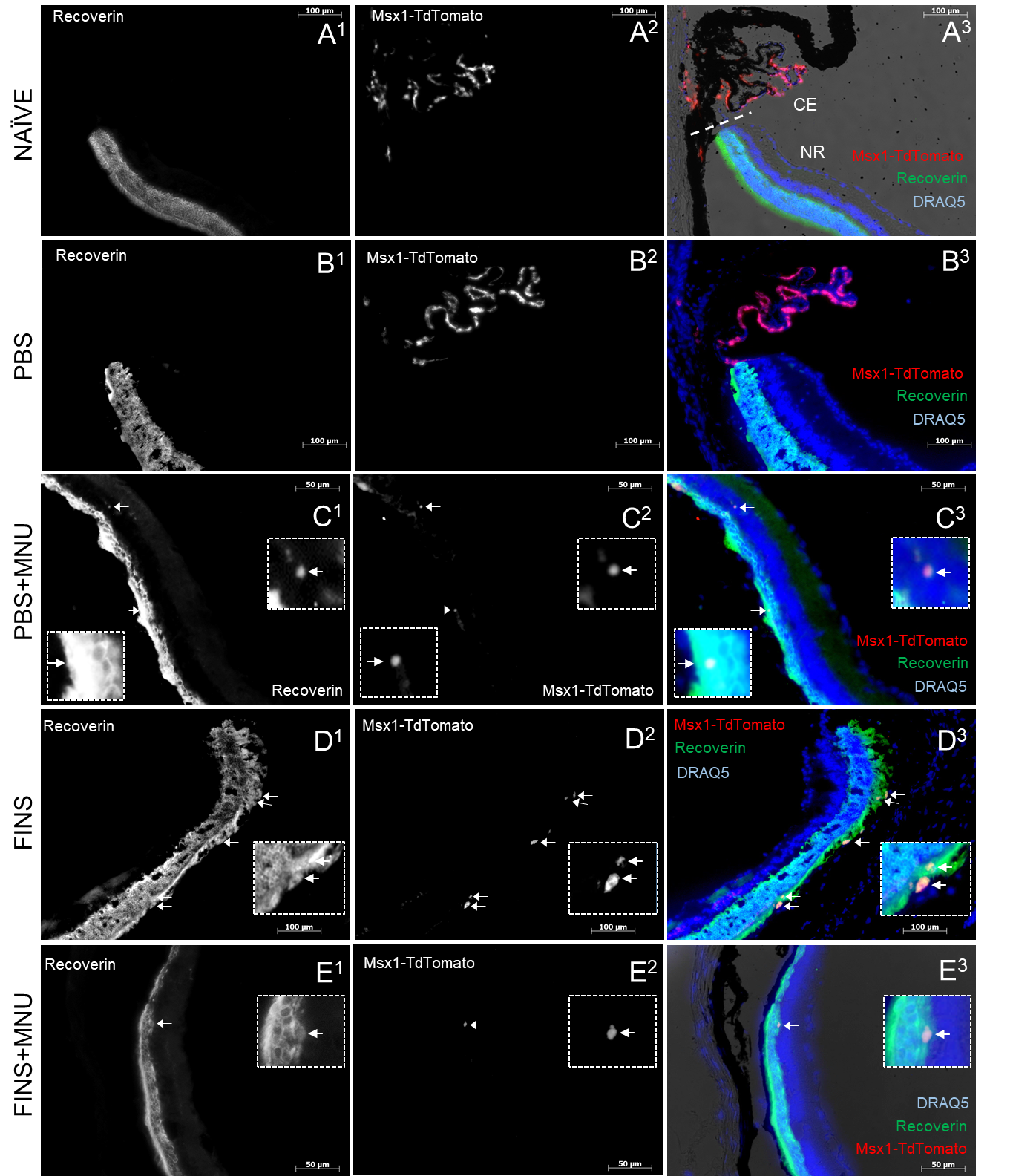


**Supplementary Figure 13. Multichannel images of the representative IHC images in Figure 6.**

**(A-D)** Brightfield and fluorescence overlay images of Recoverin immunostaining and Msx1-Cre driven tdTomato expression in eye sections from the indicated conditions. DRAQ5 stain was used to label all nuclei. White arrows indicate *Msx1*-tdTomato^+^Recoverin^+^ co-labeled cells. Dashed line box indicates high magnification inset. Straight dashed line indicates ciliary epithelium (CE) neural retina (NR) border. 10µm-thick sections.

Groups included: Naïve control = no injection; PBS = intravitreal PBS; PBS+MNU = intravitreal PBS and i.p. MNU; FINS = intravitreal FINS; FINS+MNU = intravitreal FINS and i.p. MNU. FINS = FGF2 + Insulin + Noggin + anti-sFRP2 combined intravitreal injection.

**
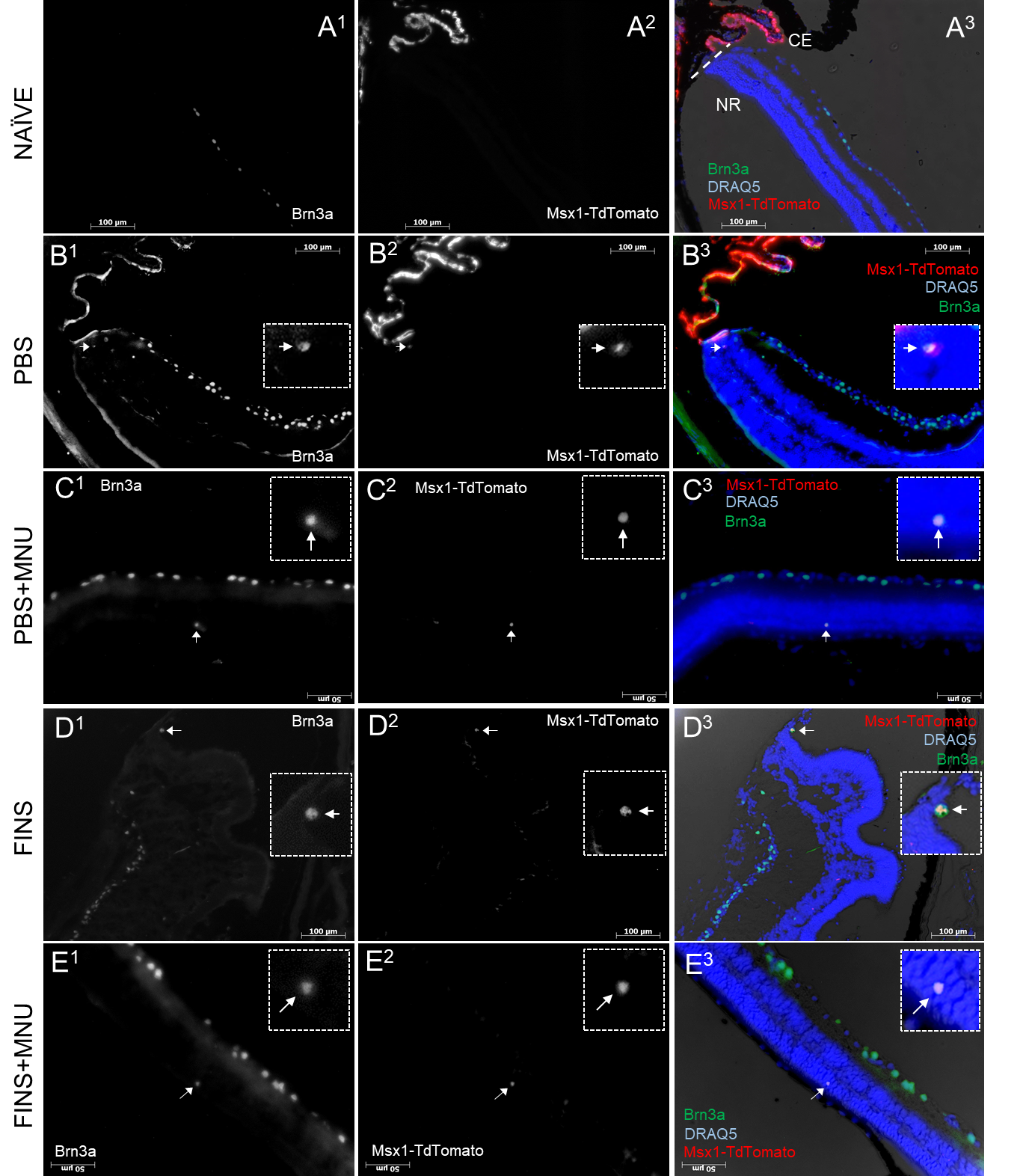
**

**Supplementary Figure 14.** Multichannel images of the representative IHC images in Figure 6.

**(A-D)** Brightfield and fluorescence overlay images of Brn3a immunostaining and Msx1-Cre driven tdTomato expression in eye sections from the indicated conditions. DRAQ5 stain was used to label all nuclei. White arrows indicate *Msx1*-tdTomato^+^Brn3a^+^ co-labeled cells. Dashed line box indicates high magnification inset. Straight dashed line indicates ciliary epithelium (CE) neural retina (NR) border. 10µm-thick sections.

Groups included: Naïve control = no injection; PBS = intravitreal PBS; PBS+MNU = intravitreal PBS and i.p. MNU; FINS = intravitreal FINS; FINS+MNU = intravitreal FINS and i.p. MNU. FINS = FGF2 + Insulin + Noggin + anti-sFRP2 combined intravitreal injection.


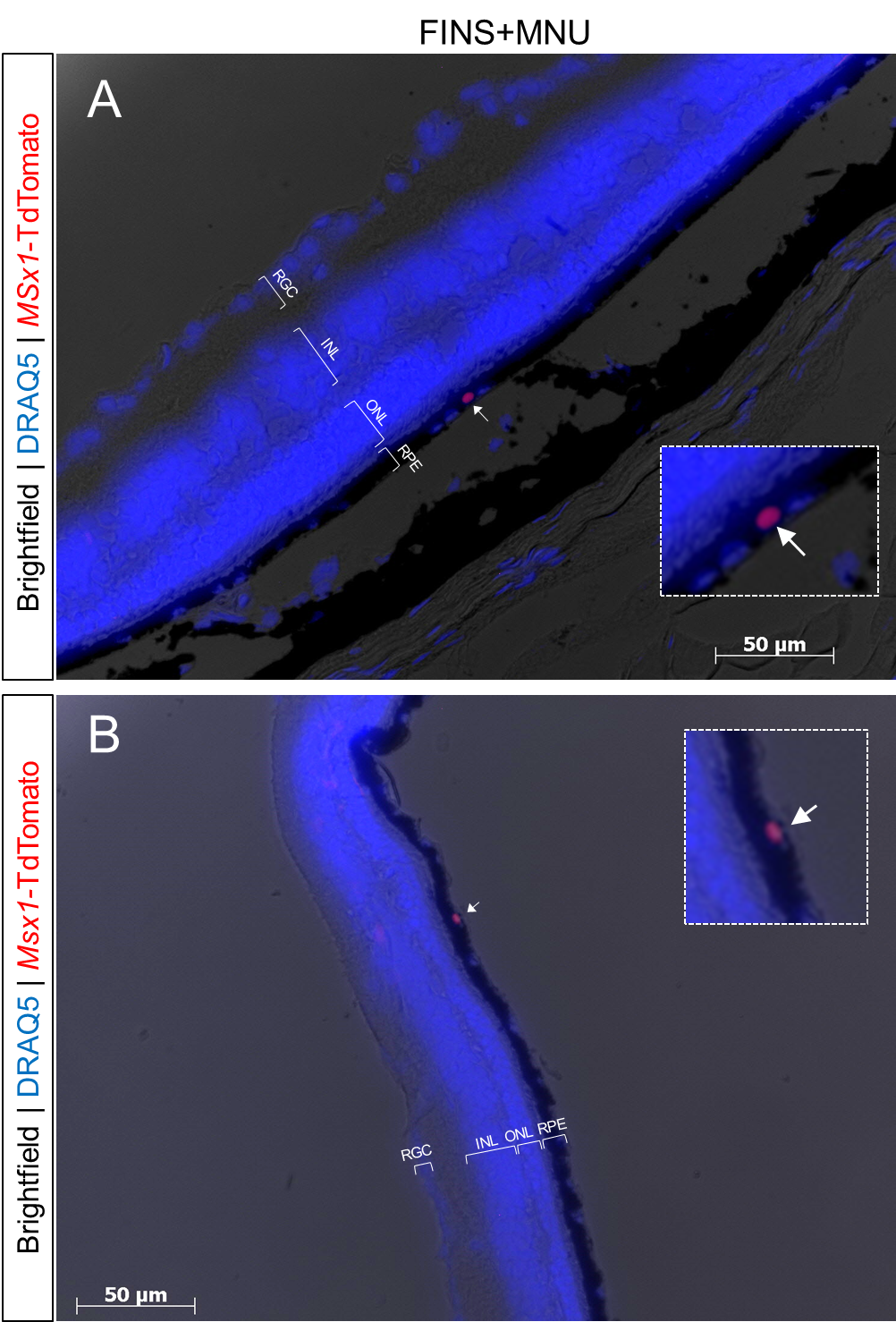


**Supplementary Figure 15. *Msx1*-tdTomato+ cells were detected in the RPE layer at very low frequency.**

**(A-B)** Msx1-tdTomato^+^ CE cells in the RPE layer of MNU injured eyes treated with FINS.

DRAQ5 was used to label nuclei. Dashed line box indicates high magnification inset. Dashed line box indicates high magnification inset. 10µm thick sections. RGC = retinal ganglion cell layer, INL = inner nuclear layer, ONL = outer nuclear layer, RPE = retinal pigmented epithelium layer.

FINS+MNU = intravitreal FINS and i.p. MNU. FINS = FGF2 + Insulin + Noggin + anti-sFRP2 combined intravitreal injection.


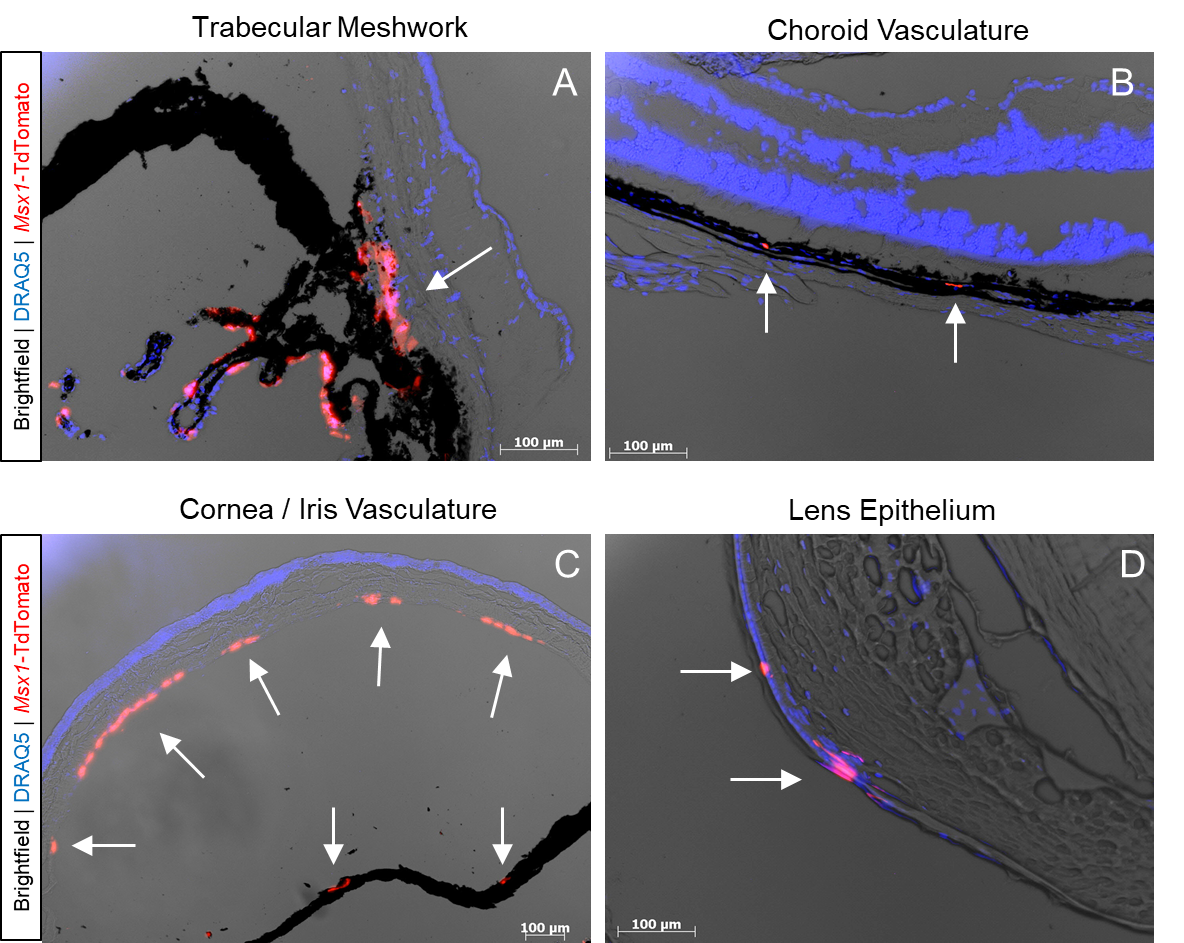


**Supplementary Figure 16. Inducible Msx1-Cre^ERT2^ mouse lineage labeling marks numerous tissues in the adult mouse eye.**

**(A**) tdTomato expression in the ciliary epithelium and the trabecular meshwork (white arrow).

**(B)** tdTomato expression in the choroid vasculature (white arrows).

**(C)** tdTomato expression in the corneal endothelium (up arrows) and iris vasculature (down arrows).

**(D)** tdTomato expression in epithelial cells of the lens (white arrows).

10µm-thick sections. DRAQ5 was used to label all nuclei.

**Table S1.** Detailed primer sequence list

| **Primer** | **Sequence (5’🡪3’)** |
| --- | --- |
| Msx1- F | GGCTGTCTCGAGCTGCGGCTGGAGGG |
| Msx1- R | CCATGGCGGTTGCGGTGGCCGCAGC |
| Cre-R | GCTGGATAGTTTTACTGCCAGACCGCGCGCC |
| pCAG-F (stop sequence evaluation) | GCAACGTGCTGGTTATTGTG |
| tdTomato-R (stop sequence evaluation) | TCTTTGATGACGGCCATGT |
| WT tdTomato-F | AAGGGAGCTGCAGTGGAGTA |
| WT tdTomato-R | CCGAAAATCTGTGGGAAGTC |
| Mut tdTomato-F | GGCATTAAAGCAGCGTATCC |
| Mut tdTomato-R | CTGTTCCTGTACGGCATGG |
